# α-Synuclein-containing erythrocytic extracellular vesicles: essential contributors to hyperactivation of monocytes in Parkinson’s disease

**DOI:** 10.1186/s12974-022-02413-1

**Published:** 2022-02-22

**Authors:** Zongran Liu, Robin Barry Chan, Zhijian Cai, Xiaodan Liu, Yufeng Wu, Zhenwei Yu, Tao Feng, Ying Yang, Jing Zhang

**Affiliations:** 1 Department of Pathology, Peking University Health Science Center, Beijing, 100191 China; 2 AliveX Biotech, Shanghai, 200030 China; 3 School of Basic Medicine, Zhejiang University, Hangzhou, 310002 Zhejiang China; 4 Department of Laboratory Medicine, Peking University Third Hospital, Peking University Health Science Center, Beijing, China; 5 Beijing Neurosurgical Institute, Capital Medical University, Beijing, 100050 China; 6 Department of Neurology, TianTan Hospital, Capital Medical University, Beijing, 100050 China; 7 Department of Pathology, Zhejiang University School of Medicine and First Affiliated Hospital, Hangzhou, 310002 Zhejiang China; 8 National Health and Disease Human Brain Tissue Resource Center, Zhejiang University, Hangzhou, 310002 Zhejiang China

**Keywords:** Extracellular vesicles, RBC, α-Synuclein, Parkinson’s disease, Monocytes, LRRK2

## Abstract

**Background:**

Immune system dysfunction, including higher levels of peripheral monocytes and inflammatory cytokines, is an important feature of Parkinson’s disease (PD) pathogenesis, although the mechanism underlying the process remains to be investigated. In the central nervous system, it is well-known that α-synuclein (α-syn), a key protein involved in PD, activates microglia potently, and it is also reported that α-syn exists in the peripheral system, especially in erythrocytes or red blood cells (RBC) at exceedingly high concentration. The current study focused on the possibility that RBC-derived α-syn mediates the sensitization of peripheral monocytes in PD patients.

**Methods:**

The hyperactivation of monocytes was assessed quantitatively by measuring mRNA levels of typical inflammatory cytokines (including IL-1β, IL-6 and TNF-α) and protein levels of secreted inflammatory cytokines (including pro-inflammatory cytokines: IL-1β, IL-6, TNF-α, IL-8, IFN-γ, IL-2, and IL-12p70 and anti-inflammatory cytokines: IL-4, IL-10, and IL-13). Western blot, nanoparticle tracking analysis and electron microscopy were used to characterize RBC-derived extracellular vesicles (RBC-EVs). Inhibitors of endocytosis and leucine-rich repeat kinase 2 (LRRK2), another key protein involved in PD, were used to investigate how these two factors mediated the process of monocyte sensitization by RBC-EVs.

**Results:**

Increased inflammatory sensitization of monocytes was observed in PD patients and PD model mice. We found that α-syn-containing RBC-EVs isolated from PD model mice or free form oligomeric α-syn induced the inflammatory sensitization of THP-1 cells, and demonstrated that endocytosis was a requirement for this pathophysiological pathway. Furthermore, the hyperactivation of THP-1 cells induced by RBC-EVs was associated with increased LRRK2 production and kinase activity. The phenomenon of inflammatory sensitization of human monocytes and increased LRRK2 were also observed by the treatment of RBC-EVs isolated from PD patients.

**Conclusions:**

Our data provided new insight into how hyperactivation of monocytes occurs in PD patients, and identified the central role played by α-syn-containing RBC-EVs in this process.

**Supplementary Information:**

The online version contains supplementary material available at 10.1186/s12974-022-02413-1.

## Background

Parkinson’s disease (PD) is a neurodegenerative disease clinically characterized by motor, e.g., resting tremor, bradykinesia, rigidity, and postural instability [1, 2], and non-motor symptoms [3]. The main pathological features of PD include the loss of dopaminergic neurons in the substantia nigra and the presence of Lewy bodies in surviving neurons [3, 4]. It has been well-demonstrated that α-synuclein (α-syn), one of the major components in Lewy bodies, exists not only in the central nervous system (CNS), but is also widespread in blood, especially in erythrocytes or red blood cells (RBCs) [5, 6]. Previously, we have demonstrated that RBCs are capable of secreting α-syn-containing extracellular vesicles (EVs) and these RBC-derived EVs (RBC-EVs) can cross the blood–brain barrier (BBB) and deposit α-syn in astrocytes and microglia [7, 8], resulting in astrocytic dysfunction and microglial activation.

In addition to impairment of motor and non-motor functions and loss of dopamine neurons, inflammation in the CNS is a salient feature of PD pathogenesis [9–11]. Although the role of neuroinflammation is not clear, microglial activation coupled with increased inflammatory cytokines and infiltration of immune cells in the CNS have been reported in PD [12–19]. Besides CNS manifestations, PD patients also frequently experience clinical manifestations that are closely linked to dysfunction of the peripheral immune system [20]. For example, in the serum of PD patients, higher levels of inflammatory cytokines have been observed, indicating the existence of peripheral inflammation [21–23]. In addition, the peripheral blood monocytes, enriched in PD patients compared to healthy controls, can secrete more inflammatory cytokines after external stimulation of bacterial endotoxin lipopolysaccharide (LPS), suggesting hyperactivation of the immune system in PD patients [24, 25]. Interestingly, basal and LPS-induced levels of cyto/chemokines in peripheral blood mononuclear cells are significantly correlated with the severity of PD symptoms [26]. Yet, the mechanisms underlying peripheral inflammation remain to be characterized.

Monocytes, a subset of circulating white blood cells, are the precursors of tissue macrophages and dendritic cells (DCs), which are equipped with high phagocytic and antigen-presenting capabilities and take part in the initial innate immune response [27]. It has been shown that oligomeric α-syn can activate microglia with the production of pro-inflammatory mediators [28, 29]. Because heteromeric and oligomeric α-syn are highly abundant in RBCs [30–35] and α-syn containing RBC-EVs isolated from PD patients can pass through the BBB and activate microglia [8], it is possible that hyperactivation of monocytes in PD is associated with oligomeric α-syn containing RBC-EVs.

Mutations in the Leucine-rich repeat kinase 2 (*LRRK2*) gene have been identified as the most common genetic cause for familial PD [36]. Recent studies have discovered that hyperactivated wild-type LRRK2 may also play a role in idiopathic PD, although the mechanism involved is not entirely known [37, 38]. LRRK2 is highly expressed in monocytes, and its kinase activity is associated with immune cell activation [39, 40]. It is likely that the LRRK2 pathway takes part in the hyperactivation of monocytes in PD.

In this study, we tested the hypothesis that α-syn-containing RBC-EVs can induce inflammatory sensitization of monocytes in PD and investigated the molecular mechanisms by which this pathogenic phenomenon occurs.

## Methods

### Human subjects and clinical sample collection

The study was approved by the Institutional Review Board of Beijing Tiantan Hospital, Capital Medical University. In this study, whole blood samples from 15 patients with PD and 9 age- and sex-matched healthy controls were collected from Tiantan Hospital. All subjects underwent evaluations including medical history, physical and neurological examinations, laboratory tests, and neuropsychological assessments. Briefly, all PD patients met UK PD Society Brain Bank clinical diagnostic criteria for PD. Control subjects were in good health without any signs or symptoms suggesting cognitive impairment or neurological disease. All participants underwent detailed informed consent procedures. Demographic information is listed in Table 1 for all subjects.

**Table 1:** Characteristics of the clinical cohort of blood samples for RBC-EVs collection and monocyte isolation

Purpose	RBC-EVs collection		Monocyte isolation	
	Healthy control	PD	Healthy control	PD
Number	3	5	6	10
Sex (female/male)	1/2	2/3	3/3	5/5
Age (mean ± SD)	66 ± 6.24	64 ± 9.51	65.17 ± 4.96	68.1 ± 4.09
UPDRS3 (mean ± SD)	n/a	50.2 ± 7.79	n/a	51.5 ± 7.12

### Animals

All animal procedures were approved in accordance with Chinese Guidelines for the ethical review of laboratory animal welfare (LA2020056). PD A53T double transgenic homozygous mice (dbl-PAC-Tg (SNCA^A53T^); Snca^−/−^) and SNCA-KO α-syn homozygous knockout mice (B6; 129X1-Snca^tm1Rosl^/J) were purchased from Jackson laboratory and kept on a 12-h light–dark cycle with ad libitum food and water. Age-matched wild-type mice (WT) were selected as the control group. In this study, 7-month-old, male mice were employed for the isolation of circulating monocytes and RBC-EVs.

### RBC-EVs isolation

RBC-EVs were isolated from cultured human and mouse RBCs according to a previous article with minor modifications [8]. Briefly, human and mouse RBCs were separated from whole blood and cultured in RPMI1640 culture medium containing 25 mM HEPES at 37 °C with a humidified atmosphere of 5% CO_2_ for 48 h. Then, culture medium was collected and centrifuged at 1500×*g* for 10 min to remove RBCs, followed by filtering the supernatant through 0.22-μm filters. Subsequent filtrate was centrifuged in an ultracentrifuge (Beckmann) at 140,000 × g for 2 h to collect EVs. The collected pellets were washed with ice cold PBS and centrifuged again at 140,000×*g* for 2 h. Finally, EVs were resuspended and collected using ice cold PBS for further experiments.

### Cell culture

THP-1 cells (ATCC #TIB-202) were maintained in RPMI1640 medium with 2 mM L-glutamine and 25 mM HEPES, supplemented with 10% fetal bovine serum.

For immune sensitization experiments, THP-1 cells were cultured with 200 μg RBC-EVs from different sources for 24 h (baseline) and then stimulated with 200 ng/mL bacterial lipopolysaccharide (LPS; Sigma, L2630; ≥ 500,000 Endotoxin Units/mg) for 24 h. THP-1 cells were collected for total RNA extraction and culture media were collected for inflammatory cytokines detection, at baseline and after LPS treatment.

For endocytosis inhibitor experiments, THP-1 cells were cultured with inhibitors for 4 h, followed by the addition of RBC-EVs. After 24-h incubation, 200 ng/mL LPS was applied for THP-1 stimulation for 24 h. Total RNA and culture media were collected for inflammatory cytokines detection.

### Monocyte isolation

Circulating monocytes in mice and human blood were isolated by peripheral blood monocytes cells separation medium (Solarbio, P5290, P5230, Beijing, China) and maintained in RPMI1640 medium with 2 mM L-glutamine and 25 mM HEPES, supplemented with 10% fetal bovine serum.

For immune sensitization experiments, circulating monocytes were cultured with 200 μg RBC-EVs from different groups for 24 h (baseline) and then stimulated with 200 ng/mL bacterial LPS for 24 h. Monocytes were collected for total RNA extraction and culture media were collected for inflammatory cytokines detection, at baseline and after LPS treatment.

### Electron microscopy

RBC-EVs isolated from different species were dropped onto the grid and then negatively stained with 2% phosphotungstic acid. The morphology of RBC-EVs was acquired by transmission electron microscopy (JEM1400 PLUS).

### EVs labelling

RBC-EVs isolated from cultured WT mice were resuspended in 1 mL PBS buffer at particle concentration of 2 × 10^10^ /mL. Subsequently, 1 mM DiI or DiO stock solution (Coolaber, SL7910, SL7920) was added into the RBC-EV suspension at final concentration of 10 μM to label the RBC-EV for 30 min at room temperature. To remove the excess DiI or DiO dye, the labelled RBC-EV suspension was filtered thrice through Amicon® Ultra-centrifugal filter devices (cutoff MW 30 kDa, Millipore Corporation, Billerica, MA, USA). Finally, the labelled RBC-EVs were collected for further experiments or stored at –80 °C.

### Nanoparticle tracking analysis

The particle concentration and size distribution of RBC-EVs were detected by NTA instrument (NS300; Nanosight). RBC-EVs were first diluted by filtered PBS at 1:100 to optimize the number of particles and injected into the sample sink. The movement of each particle was video captured in triplicate of 60 s. Analysis was performed by NTA 3.2 software (Nanosight, Amesbury, UK).

### Real-time quantitative PCR

Total RNA was isolated from THP-1 cells and monocytes by TRIZOL™ reagent (Invitrogen, 15,596,018) according to the manufacturer's instruction. Following the extraction of RNA, cDNA was reverse transcribed from 2 mg total RNA by RT Master Mix Kit with gDNase (MCE, HY-K0511). The mRNA level of target gene in acquired cDNA was determined by real-time PCR with PowerUp SYBR® Green Master Mix (applied biosystem, A25742). *ACTB* was used as the internal reference for normalization. The primers used are presented in Table 2. All results were analyzed using 2^−△△Ct^ and were presented as mean ± SEM.

**Table 2:** Primers used in qPCR

Gene	Forward primer (5′ to 3′)	Reverse primer (5′ to 3′)
*Il1b*	TGGACCTTCCAGGATGAGGACA	GTTCATCTCGGAGCCTGTAGTG
*Il6*	TACCACTTCACAAGTCGGAGGC	CTGCAAGTGCATCATCGTTGTTC
*Tnf*	GGTGCCTATGTCTCAGCCTCTT	GCCATAGAACTGATGAGAGGGAG
*Actb*	CATTGCTGACAGGATGCAGAAGG	TGCTGGAAGGTGGACAGTGAGG
*IL1b*	CCACAGACCTTCCAGGAGAATG	GTGCAGTTCAGTGATCGTACAGG
*IL6*	AGACAGCCACTCACCTCTTCAG	TTCTGCCAGTGCCTCTTTGCTG
*TNF*	CTCTTCTGCCTGCTGCACTTTG	ATGGGCTACAGGCTTGTCACTC
*LRRK2*	AGCAAGGGACAGGCTGAAGTTG	GCAGGCTTTGCGTTGCTTCTCA
*ACTB*	CACCATTGGCAATGAGCGGTTC	AGGTCTTTGCGGATGTCCACGT

### Western blotting

Proteins were extracted from THP-1 cells and monocytes using cell lysis buffer (RIPA, HARVEYBIO, C1503) containing protease inhibitor cocktail (Sigma) and phosphatase inhibitor PhosSTOP™ (Roche, 4,906,837,001). BCA Protein Assay Kit (Applygen, P1511) was applied to determining the protein concentration of RBC-EVs and THP-1 monocytes according to the manufacturer's instruction. A total of 20 μg RBC-EVs or 30 μg THP-1 lysates were loaded to 10% PAGE gels for electrophoresis. Separated proteins were blotted to PVDF membrane (Merck Millipore, 4515). Then the membranes were blocked in TBST buffer (Applygen, B1009) containing 5% BSA (Amresco, A-0332) for 1 h at room temperature and incubated with diluted primary antibody overnight at 4 °C. Antibodies used included Alix (1:1000, Merck, Abc40), TSG101 (1:1000, abcam, ab133586), LRRK2 (1:5000, Abcam, ab133474), p-Rab10 (1:1000, Abcam, ab230261), Rab10 (1:1000, Abcam, ab237703), and β-actin (1:1000, Zsgb-bio, TA-09). The membranes were washed three times by TBST buffer on the second day, followed by incubation with secondary antibody for 1 h at RT. Protein bands were developed by enhanced chemiluminescence reagents (Millipore, WBKLS0100) and detected by Chemi doc™ Imaging System (Bio-Rad, Hercules CA, USA).

### Flow cytometry for RBC-EV uptake assay

THP-1 monocytes pre-treated with or without endocytosis inhibitors for 30 min were incubated with 2 × 10^9^ DiO-labelled RBC-EVs for another 4 h. Then, monocytes were collected and washed for analysis using BD FACS Calibur Flow Cytometer (BD Biosciences, Franklin Lakes NJ, USA) to determine the uptake of RBC-EVs in monocytes. Endocytosis inhibitors included Me-β-CD (200 μM, MCE, HY-101461), Amiloride (1 mM, MCE, HY-B0285A), Dynasore (50 μM, Selleck, S8047), and Nocodazole (40 ng/mL, MCE, HY-13520). The inhibition rate was calculated as the following equation:

$$\text{Inhibition rate}\left(\text{\%}\right)\text{=} \, \text{1} - \frac{\text{The number of monocytes containing DiO signal with inhibitors}}{\text{The number of monocytes containing DiO signal with vehicle} \, {\text{control}}}$$

### Laser confocal microscopy for RBC-EV endocytosis assay

2 × 10^9^ Dil labelled RBC-EVs were added into THP-1 monocytes pre-treated with or without endocytosis inhibitors for 30 min and incubated for 4 h. The cell nucleus was stained by Hoechst33258 (Solarbio) for 15 min. Images were acquired using Leica TCS SP8 confocal system.

### Meso scale discovery multiplexed immunoassays

The detection and quantification for total and aggregated α-syn were based on our previously developed and validated assay [35]. Briefly, antibody against α-syn, MJFR-1 (ab138501, Abcam, only recognizes humanized α-syn) and antibody against conformation specific α-syn filaments, MJFR-14 (ab209538, Abcam, recognizes the structure of α-syn oligomers from both mouse and human species) were, respectively, biotinylated, linker conjugated, and coated onto standard 96-well U-Plex plates (Meso scale discovery). After washing three times with 150 μl Washing Buffer, 50 µl of diluted sample (diluted by Diluent 35 at dilution ratio of 1:5) and calibrator (recombinant α-syn, Sino biological, and oligomeric α-syn, Proteos) were loaded to the immunoassay plate, which was incubated overnight at 4 °C on a shaker at 600 rpm. Next, the plate was washed three times using washing buffer followed by the addition of sulfo-TAG-labelled anti-α-syn antibody (BD42) and incubated at room temperature for 1 h on a shaker at 600 rpm. Finally, 150 μL 2 × read buffer T was added into each well and plates were analyzed in a Quickplex SQ 120 (MSD, USA). Data analysis was performed with the MSD Discovery Workbench 3.0 Data Analysis Toolbox. Total α-syn concentrations were normalized to the total protein levels in RBC-EV, and are shown in unit of pg (total α-syn)/mg (total protein). Oligomeric α-syn concentrations were normalized to the corresponding total α-syn levels, and are shown in units of pg (oligo α-syn)/mg (total protein).

The inflammatory cytokines in culture medium were detected by 10-Vplex plates (containing IFN-γ, IL-10, IL-12p70, IL-13, IL-1β, IL-2, IL-4, IL-6, IL-8, and TNFα, Meso scale discovery) according to the manufacturer's instruction. Plates were analyzed in a Quickplex SQ 120 (MSD, USA). Data analysis was performed with the MSD Discovery Workbench 3.0 Data Analysis Toolbox.

### Statistical analysis

Statistical analyses were performed using Prism 9.0 (GraphPad, USA). The statistical significance was assessed by *t* test or ANOVA analysis, followed with Tukey–Kramer’s post-hoc test, Dunn’s post-hoc test or Bonferroni’s post hoc test for multiple comparisons.

## Results

### Sensitization of monocytes in PD

**Fig. 1:**
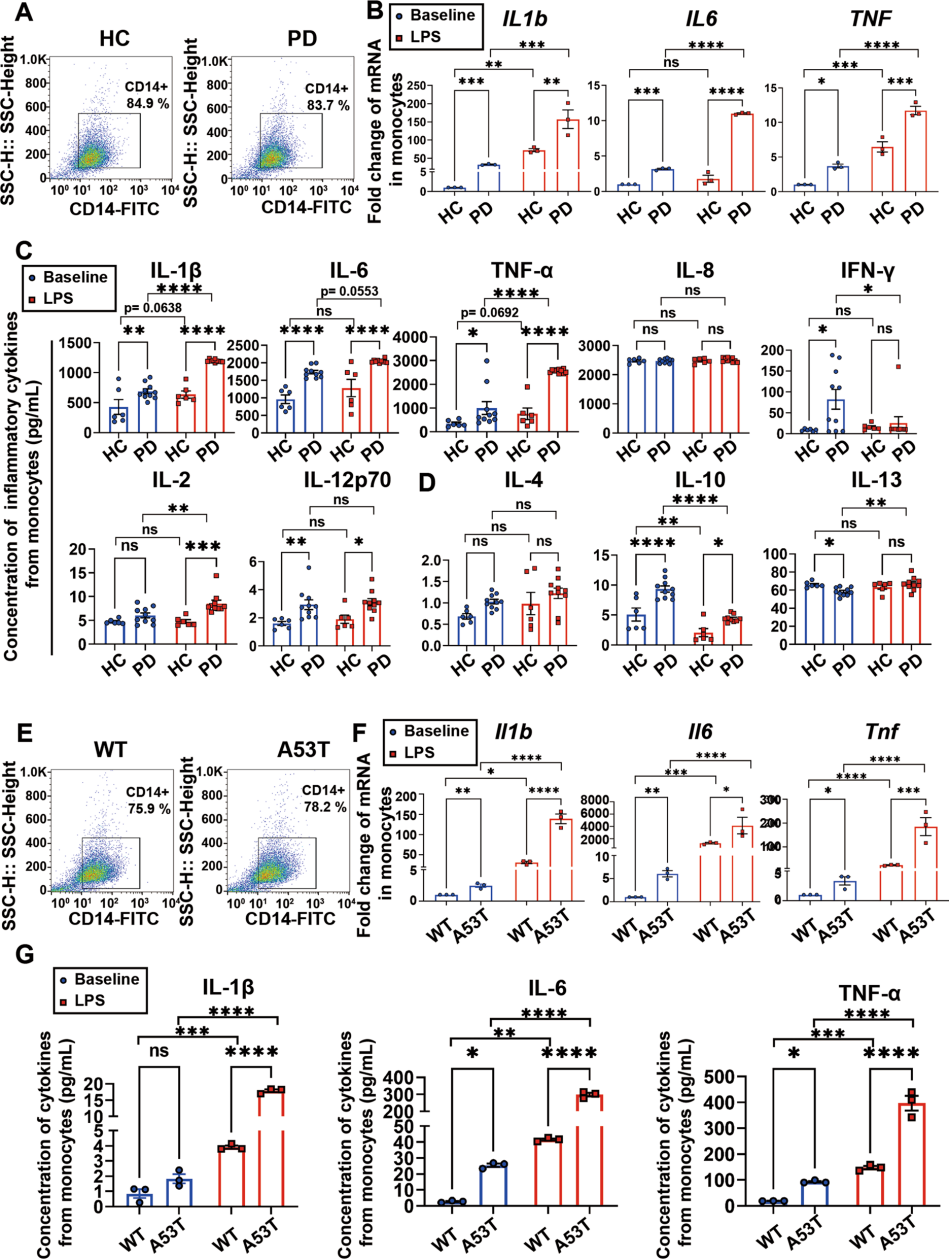
Characterization of monocytes in PD patients and mice. **A** Purity of monocytes isolated from PD patients and healthy controls were evaluated by flow cytometry. The monocytes were labelled with CD14-FITC. **B** Quantitative analysis of *IL1b*, *IL6* and *TNF* mRNA levels using qPCR, in human monocytes at resting state (blue) and stimulated with LPS for 24 h (red). *N* = 3 independent pooled human samples in each group. **C** Quantitative analysis of pro-inflammatory cytokines IL-1β, IL-6, TNF-α, IL-8, IFN-γ, IL-2, and IL-12p70, and **D** anti-inflammatory cytokines IL-4, IL-10, and IL-13 using MSD, released by human monocytes at resting state (blue) and stimulated with LPS for 24 h (red). *N* = 6 independent human samples in healthy control group and *N* = 10 independent human samples in PD group. **E** Purity of monocytes isolated from A53T mice and WT mice were evaluated by flow cytometry. The monocytes were labelled with CD14-FITC. **F** Quantitative analysis of *Il1b*, *Il6* and *Tnf* mRNA levels using qPCR, in mouse monocytes at resting state (blue) and stimulated with LPS for 24 h (red). *N* = 3 pooled independent animals in each group. **G** Quantitative analysis of cytokines IL-1β, TNF-α and IL-6 using ELISA, released by mouse monocytes at resting state (blue) and stimulated with LPS for 24 h (red). *N* = 3 pooled independent animals in each group. Values are means ± S.E.M, two-way ANOVA with Bonferroni’s post hoc test. *, *P* < 0.05; **, *P* < 0.01; ***, *P* < 0.001; ****, *P* < 0.0001

To investigate the possibility of hyperactivation of monocytes in PD, we started with examining the expression levels of typical inflammatory cytokines of the monocytes isolated from PD patients and healthy controls at baseline and following 24 h LPS stimulation. The purity of CD14 + monocytes was around 84% (Fig. 1A). The mRNA levels of typical pro-inflammatory cytokines, IL-1β, IL-6 and TNF-α, were significantly higher in monocytes of PD patients than healthy controls (Fig. 1B). The protein levels of released inflammatory cytokines (including pro-inflammatory cytokines: IL-1β, IL-6, TNF-α, IL-8, IFN-γ, IL-2, and IL-12p70 and anti-inflammatory cytokines: IL-4, IL-10, and IL-13) were measured by a commonly utilized MSD platform. In the absence of external stimuli, most of the detected inflammatory cytokine levels, except for IL-8, IL-2, IL-4 and IL-13, were higher in the monocytes of PD patients than healthy controls (Fig. 1C, D). To further evaluate the sensitization of monocytes in PD patients, monocytes were stimulated by LPS; following 24 h stimulation, the mRNA levels of *IL1b, IL6* and *TNF* and the protein levels of pro-inflammatory cytokines, especially IL-1β, IL-6, TNF-α and IL-2 became significantly higher in monocytes of PD patients than healthy controls (Fig. 1B, C), indicating the hyperactivation of monocytes in PD patients. The concentration of inflammatory cytokines in monocytes after LPS stimulation was normalized to the concentration of inflammatory cytokines in monocytes at resting state. As shown in Additional file 1: Fig. S1, LPS stimulation can elicit increased production of pro-inflammatory cytokines (IL-1β, TNF-α and IL-2) in monocytes of PD patients, which further indicated increased immune sensitization in PD. To explore the specific mechanism regulating the hyperactivation of monocytes in PD, we analyzed monocytes obtained from the dbl-PAC-Tg (SNCA^A53T^) and Snca^−/−^ double transgenic PD A53T model mice (A53T mice) [41, 42]. The purity of CD14 + mouse monocytes was approximately 75% (Fig. 1E). Subsequently, both mRNA and protein expression levels of typical pro-inflammatory cytokines, IL-1β, IL-6, and TNF-α, were examined in monocytes derived from A53T mice and WT mice. At resting state, the production of all three cytokines was higher in monocytes of A53T mice than WT mice (except for protein level of IL-1β). Following 24 h LPS stimulation, significant increases in all three pro-inflammatory cytokines were observed in the monocytes from A53T mice, compared with WT mice (Fig. 1F, G), indicating that monocytes were hyperactivated in A53T mice.

### Characterization of RBC-EVs

**Fig. 2:**
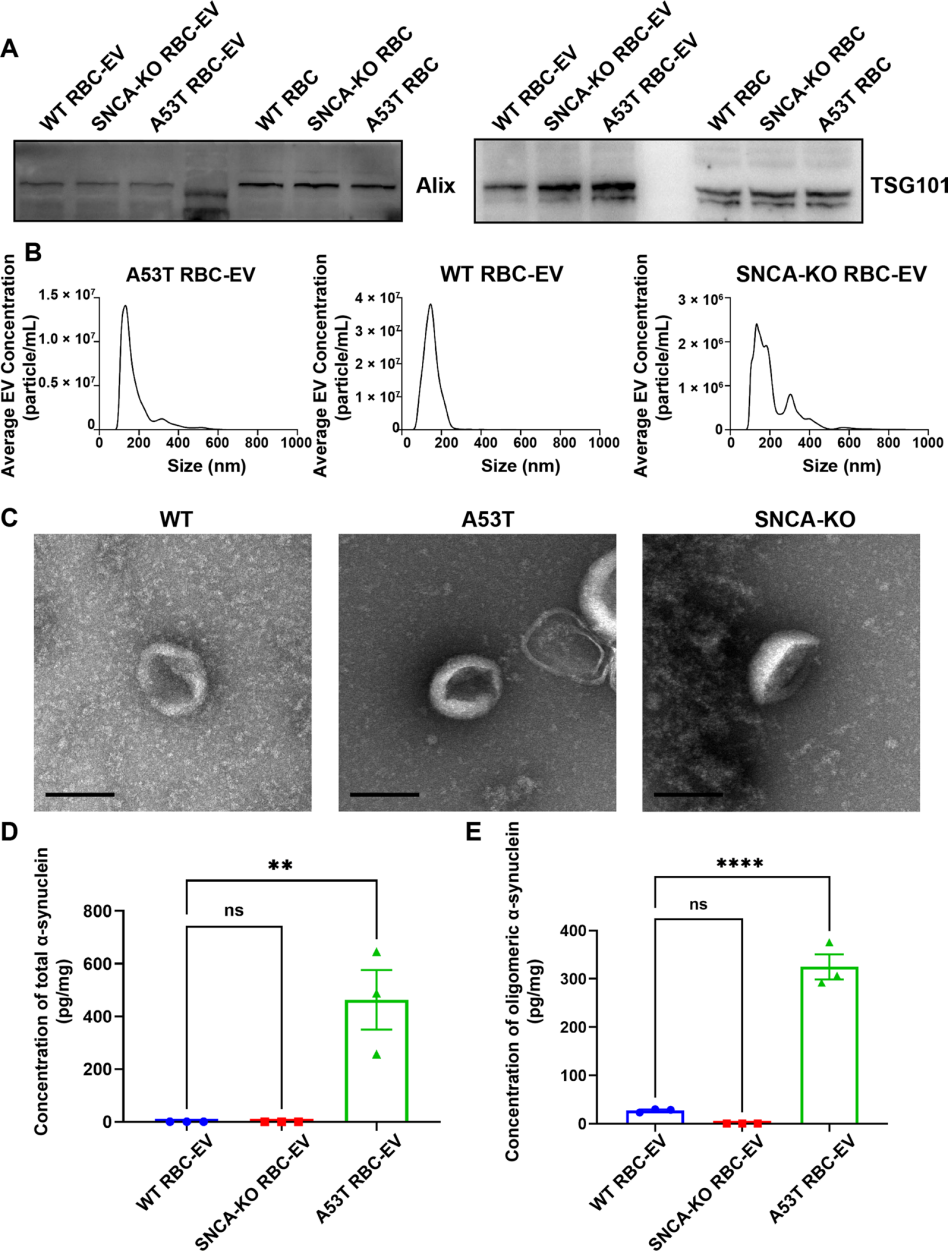
Characterization of RBC-EVs from A53T mice, WT mice and SNCA KO mice. **A** Western blot to assess the presence of EV markers Alix and TSG101 in RBC-EV lysates and RBC lysates from A53T mice, WT mice and SNCA KO mice. **B** Nanoparticle trafficking to analyze the size distribution and concentration of RBC-EVs from A53T mice, WT mice and SNCA KO mice. **C** Representative electron micrograph images of RBC-EVs from A53T mice, WT mice and SNCA KO mice. Scale bar, 500 nm. **D** Levels of total α-syn in RBC-EVs from A53T mice, WT mice and SNCA KO mice. **E** Levels of oligomeric α-syn in RBC-EVs from A53T mice, WT mice and SNCA KO mice. Values are means ± S.E.M, one-way ANOVA test. *, *P* < 0.05; **, *P* < 0.01; ***, *P* < 0.001; ****, *P* < 0.0001

We previously demonstrated that α-syn containing RBC-EVs isolated from PD patients can pass through the BBB and activate the microglia [8]. To explore potential mechanisms involved in sensitizing monocytes, RBC-EVs were isolated from cultured RBCs of the A53T, SNCA knockout (SNCA-KO) and WT mice and characterized by Western blot, NTA and TEM. As shown in Fig. 2A, the typical markers of EVs, including Alix and TSG101, were enriched in RBC-EVs from A53T, SNCA-KO and WT mice. The size distribution and particle concentration of the RBC-EVs evaluated by NTA were mainly between 70 to 200 nm in diameter (Fig. 2B). Under TEM, RBC-EVs displayed a cup-shaped bilayer membrane structure (Fig. 2C). When the level of total α-syn in EVs was measured with a well-established protocol [35], the concentration of α-syn in WT and SNCA-KO RBC EVs was below detection limit, while the mean concentration of α-syn in A53T RBC-EVs was 487.67 pg/mg. Oligomeric α-syn contained in RBC-EVs was also significantly higher in A53T mice than WT and SNCA-KO mice (A53T: 324.8 pg/mg; WT: 27.35 pg/mg; SNCA-KO: 0 pg/mg).

### α-Syn-containing RBC-EVs sensitized monocytes potently

**Fig. 3:**
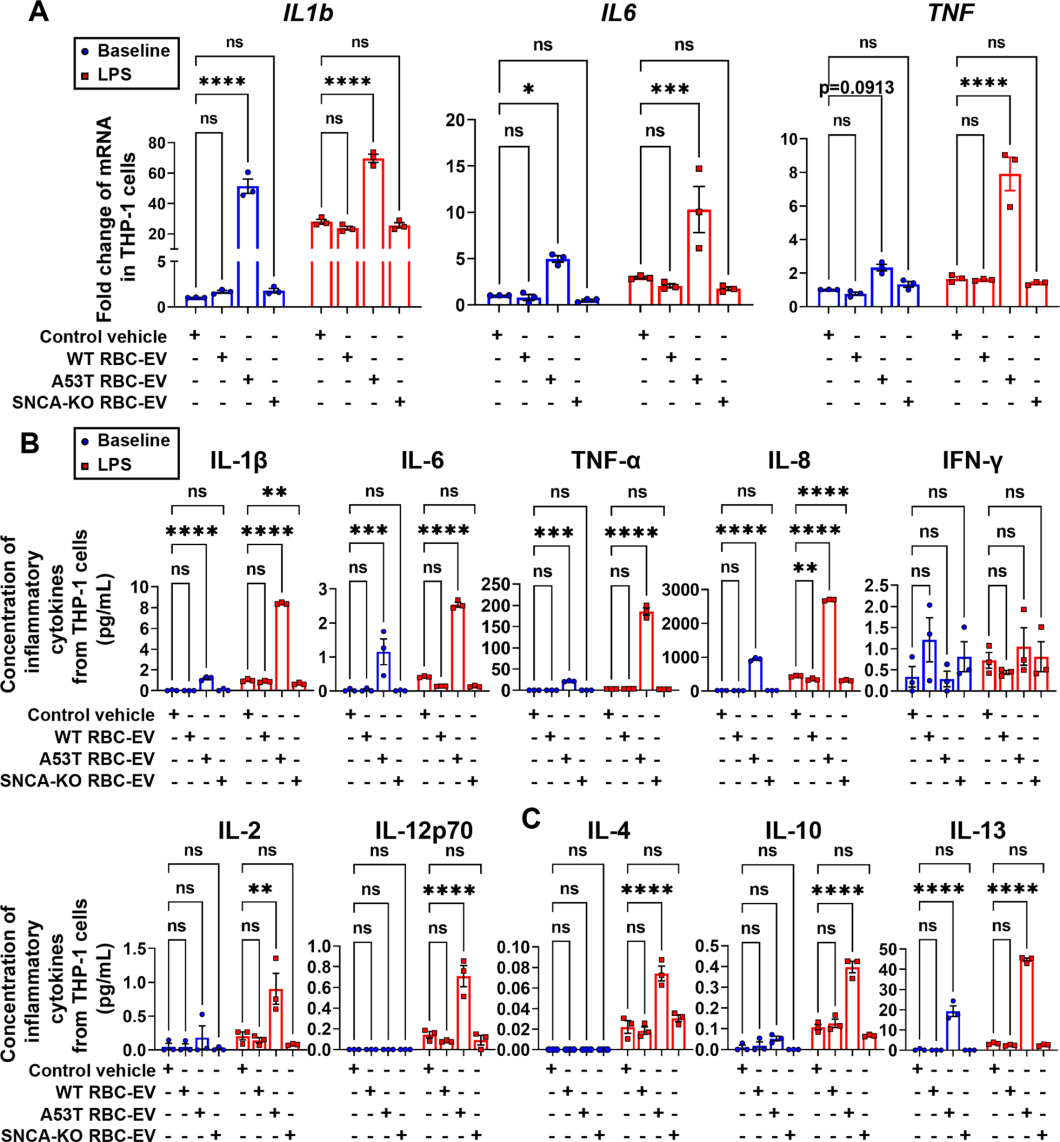
α-Syn-containing RBC-EVs from A53T mice induced immune sensitization of THP-1 cells. **A** Quantitative analysis of *IL1b*, *IL6* and *TNF* mRNA levels using qPCR, in THP-1 cells pretreated with RBC-EVs from A53T mice, WT mice or SNCA KO mice at resting state (blue) and stimulated with LPS for 24 h (red). **B** Quantitative analysis of pro-inflammatory cytokines IL-1β, IL-6, TNF-α, IL-8, IFN-γ, IL-2, and IL-12p70, and **C** anti-inflammatory cytokines IL-4, IL-10, and IL-13 using MSD, released by THP-1 cells pretreated with RBC-EVs from A53T mice, WT mice or SNCA KO mice at resting state (blue) and stimulated with LPS for 24 h (red). *N* = 3 independent experiments in each group. Values are means ± S.E.M, two-way ANOVA with Bonferroni’s post hoc test. *, *P* < 0.05; **, *P* < 0.01; ***, *P* < 0.001; ****, *P* < 0.0001

**Fig. 4:**
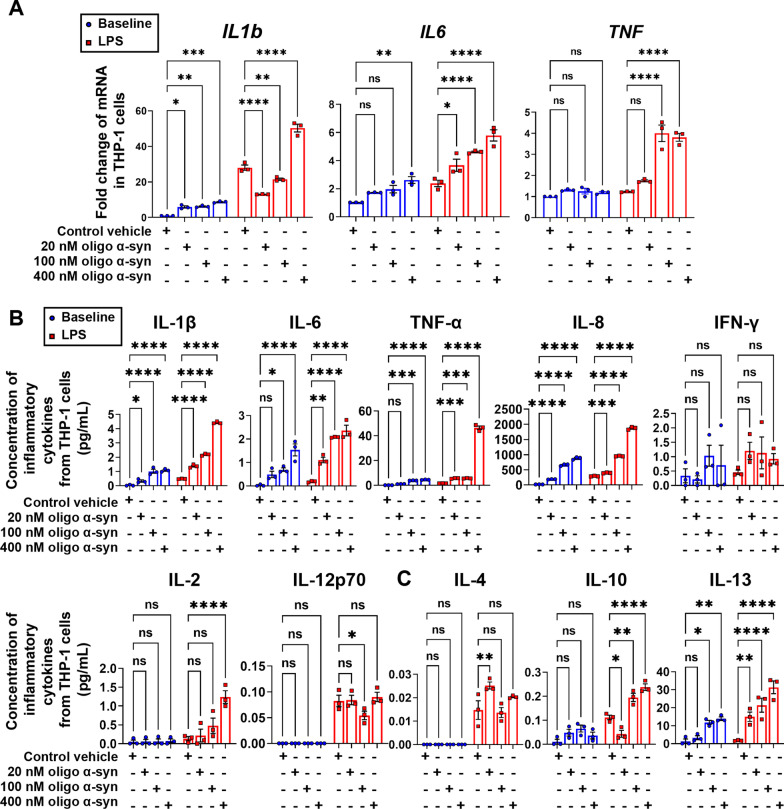
Oligomeric α-syn dose-dependently induced immune sensitization of THP-1 cells. **A** Quantitative analysis of *IL1b*, *IL6* and *TNF* mRNA levels using qPCR, in THP-1 cells pretreated with oligomeric α-syn at resting state (blue) and stimulated with LPS. **B** Quantitative analysis of pro-inflammatory cytokines IL-1β, IL-6, TNF-α, IL-8, IFN-γ, IL-2, and IL-12p70, and **C** anti-inflammatory cytokines IL-4, IL-10, and IL-13 using MSD, released by THP-1 cells pretreated with oligomeric α-syn at resting state (blue) and stimulated with LPS for 24 h (red). *N* = 3 independent experiments in each group. Values are means ± S.E.M, two-way ANOVA with Bonferroni’s post hoc test. *, *P* < 0.05; **, *P* < 0.01; ***, *P* < 0.001; ****, *P* < 0.0001

To explore the possibility of immune regulation of A53T RBC-EVs on monocytes, THP-1, a human leukemia monocytic cell line extensively used to study monocyte functions [43], was selected initially as the experimental model. As shown in Fig. 3A, the mRNA levels of *IL1b, IL6* and *TNF* in THP-1 cells treated with RBC-EVs derived from A53T mice were significantly elevated compared to those measured in THP-1 cells treated with vehicle control, while no significant increase existed in THP-1 cells treated with WT RBC-EVs or SNCA KO RBC-EVs. Alterations in the concentration of pro-inflammatory cytokines released in the culture media paralleled the mRNA results (Fig. 3B, blue bars), suggesting that A53T RBC-EVs significantly activated the THP-1 cells. Furthermore, after pre-treatment with RBC-EVs, the THP-1 cells were stimulated for 24 h with LPS to elicit an additional inflammatory response (Fig. 3A–C, red bars). These results suggest that α-syn might be a key mediator in the immune sensitization of monocytes. The hypothesis was further substantiated by exposing THP-1 cells to free oligomeric α-syn (Fig. 4) or monomeric α-syn (Additional file 1: Fig. S2), demonstrating that oligomeric α-syn significantly and dose-dependently hyperactivated monocytes.

### Endocytosis required for sensitization of monocytes induced by A53T RBC-EVs

**Fig. 5:**
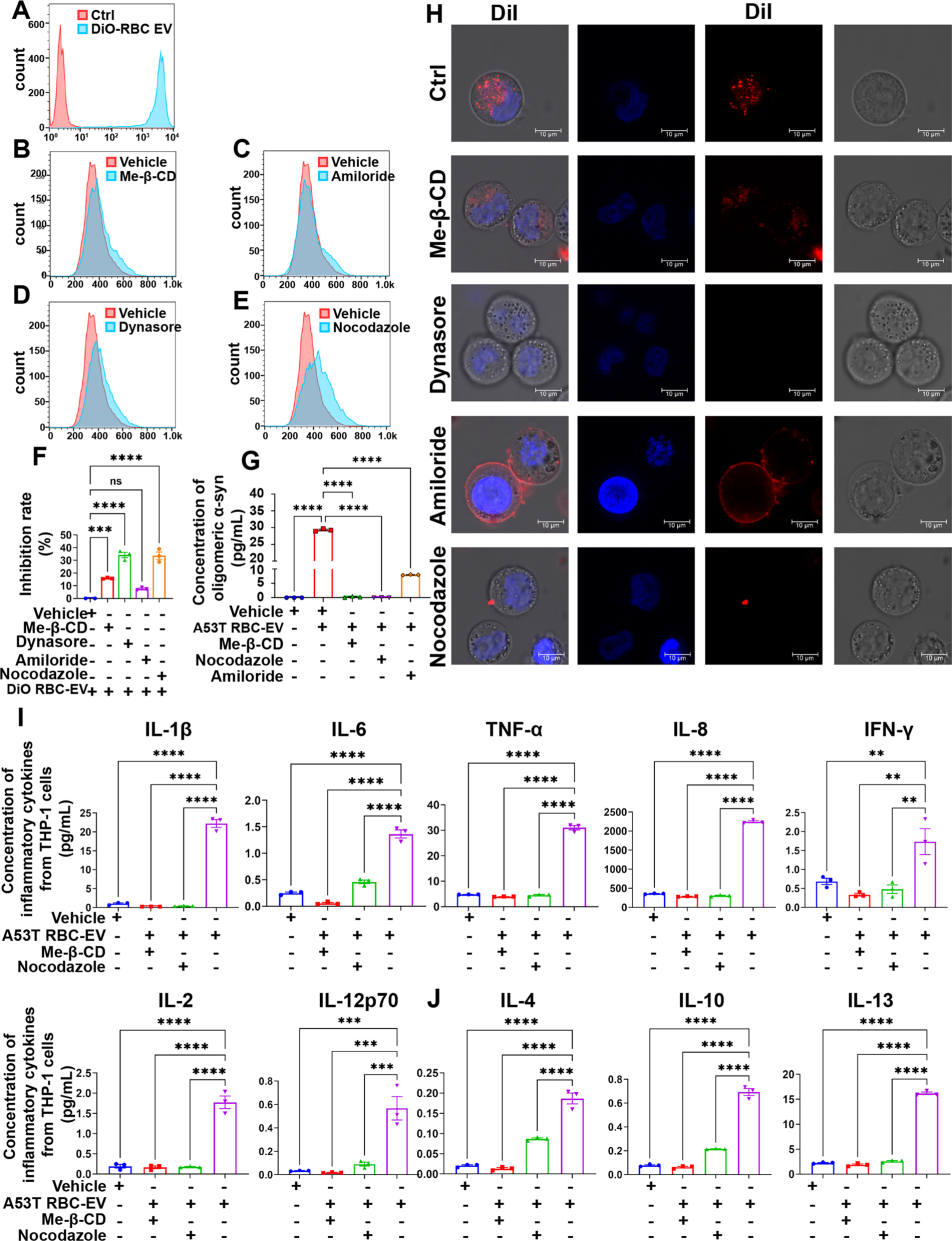
Endocytosis was involved in immune sensitization of monocytes induced by A53T RBC-EVs. **A** RBC-EVs uptake by THP-1 cells was determined by flow cytometry. Red histogram represented THP-1 cells without RBC-EVs, while blue histogram represented THP-1 cells containing RBC-EVs. The RBC-EVs were labelled with DiO. **B**–**E** Effects of endocytosis inhibitors on RBC-EVs uptake in THP-1 cells was determined by flow cytometry**.** Red histogram represented THP-1 cells co-treated with RBC-EVs and vehicle control, while blue histogram represented THP-1 cells co-treated with RBC-EVs and endocytosis inhibitor. **F** Quantitative analysis of inhibition ratio of Me-β-CD, Dynasore, Amiloride and Nocodazole. **G** Levels of oligomeric α-syn in THP-1 cells pretreated with A53T RBC-EVs, along with Me-β-CD, Nocodazole or Amiloride. **H** Representative confocal image of THP-1 cells incubated with DiI (red)-labelled RBC-EVs after treatment with Dynasore, Nocodazole Me-β-CD and Amiloride, cell nucleus was labelled by Hoechst 33258. Scale bar, 10 µm. **I** Quantitative analysis of pro-inflammatory cytokines IL-1β, IL-6, TNF-α, IL-8, IFN-γ, IL-2, and IL-12p70, and **J** anti-inflammatory cytokines IL-4, IL-10, and IL-13 using MSD, released by THP-1 cells pretreated with A53T RBC-EVs alone or with Nocodazole or Me-β-CD, followed by LPS stimulation. *N* = 3 independent experiments in each group. Values are means ± S.E.M, one-way ANOVA test. *, *P* < 0.05; **, *P* < 0.01; ***, *P* < 0.001; ****, *P* < 0.0001

To further probe the mechanisms involved in monocyte sensitization, four types of endocytosis inhibitors, specifically Me-β-CD, Amiloride, Dynasore and Nocodazole [44–47], were used to test whether internalization of RBC-EVs is necessary. By flow cytometry, we found that the RBC-EVs entered into THP-1 cells readily but this process was significantly inhibited by a 30-min treatment of four endocytosis inhibitors to different extents, with 15.7%, 7.6%, 34.4% and 33.7% inhibition for Me-β-CD, Amiloride, Dynasore and Nocodazole, respectively (Fig. 5A–F). These results were confirmed by confocal microscopy showing that abundant DiI labelled RBC-EVs can be detected in THP-1 cells treated with blank vehicle, while DiI signal was mostly confined to the plasma membrane in Amiloride treated cells, and barely observed in THP-1 cells treated with Dynasore and Nocodazole. For Me-β-CD treatment group, slight DiI signals can be detected in THP-1 cells (Fig. 5H). Next, we analyzed the effect of the four endocytosis inhibitors on monocyte hyperactivation and found that Dynasore itself significantly induced the production of pro-inflammatory cytokines TNF-α and IL-8 (Additional file 1: Fig. S3), likely attributable to the fact that Dynasore can activate the NF-κb pathway and promote the production of some pro-inflammatory cytokines [44]. Thus, Dynasore was excluded in the next series of experiments. To test the hypothesis that endocytosis of RBC-EVs is required to induce sensitization of monocytes, we pre-treated THP-1 cells with A53T RBC-EVs alone, or co-treated with Nocodazole, Me-β-CD or Amiloride for 24 h, followed by LPS stimulation. Measurement of the cytokines released by THP-1 cells revealed that Nocodazole and Me-β-CD co-treatment significantly inhibited the elevated inflammatory response of THP-1 cells induced by A53T RBC-EVs (Fig. 5I, J). To a lesser extent, Amiloride also had an inhibitory effect on THP-1 sensitization (Additional file 1: Fig. S4). To further investigate the role of α-syn in this process, we examined endocytosed oligomeric α-syn levels in THP-1 cells. As observed in Fig. 5G, around 30 pg/ml oligomeric α-syn was detected in THP-1 cells co-treated with A53T RBC-EVs and vehicle control, while no oligomeric α-syn was detectable in THP-1 cells treated with vehicle control alone, indicating the delivery of α-syn oligomers from RBC-EVs to the THP-1 cells. Compared with A53T RBC-EVs and vehicle control co-treatment group, oligomeric α-syn was hardly detected in THP-1 cells co-treated with A53T RBC-EVs and Nocodazole or with A53T RBC-EVs and Me-β-CD, indicating that Nocodazole and Me-β-CD could largely abolish the delivery of α-syn oligomers into the THP-1 cells. Taken together, these results indicate that α-syn-containing RBC-EVs can sensitize monocytes and this process is mediated by endocytosis.

### LRRK2 involved in monocyte sensitization induced by A53T RBC-EVs

**Fig. 6:**
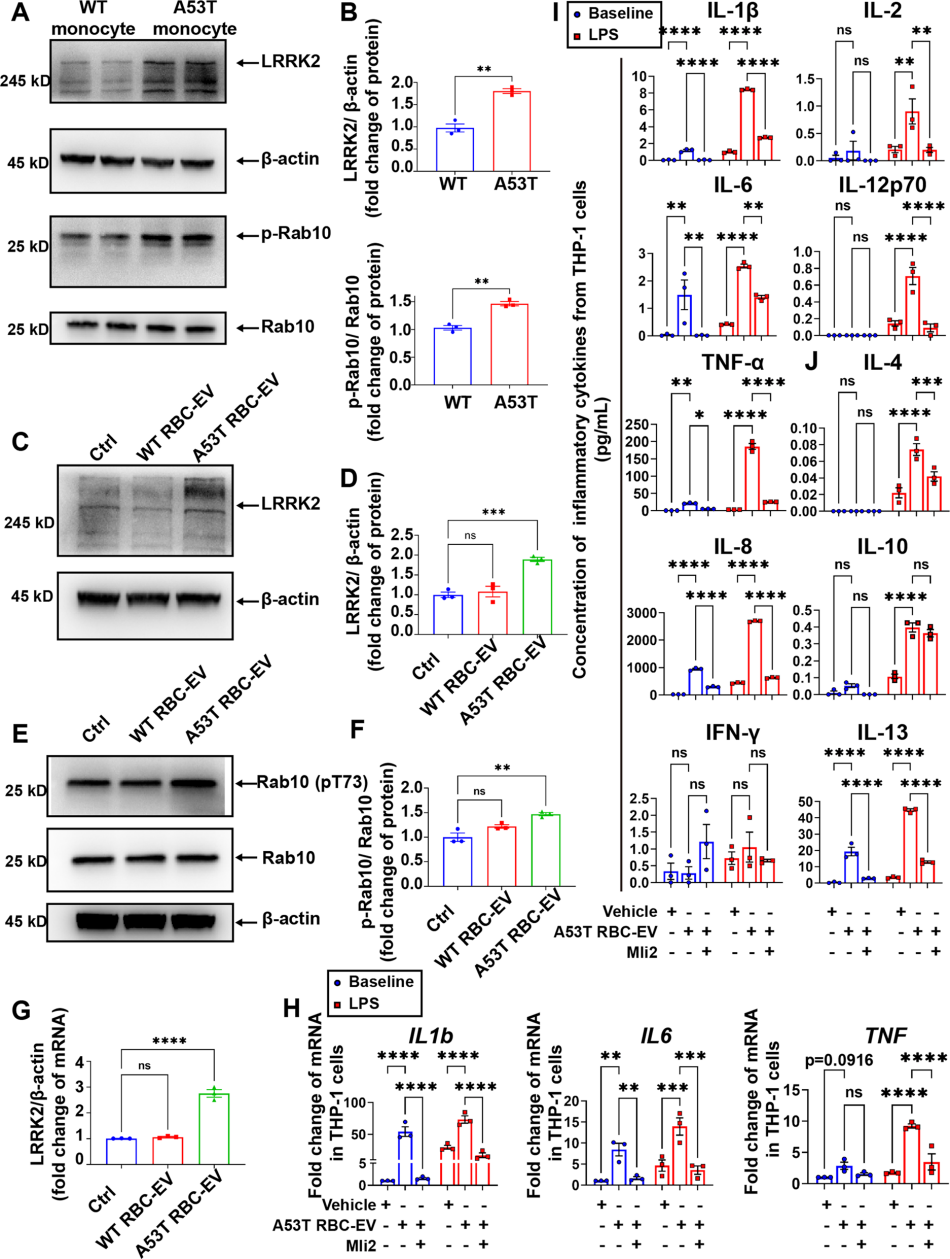
Increased LRRK2 in hyperactivated monocytes induced by A53T RBC-EVs.** A** Western blot to assess the level of LRRK2 and p-Rab10 in monocytes of A53T mice and WT mice. **B** Quantitative analysis of the level of LRRK2 and ratio of p-Rab10 to Rab10 in monocytes of A53T mice and WT mice. **C** Western blot to assess the level of LRRK2 in THP-1 cells treated with RBC-EVs from A53T mice and WT mice. **D** Quantitative analysis of the level of LRRK2 in THP-1 cells treated with RBC-EVs from A53T mice and WT mice. **E** Western blot to assess the level of Rab10 in THP-1 cells treated with RBC-EVs from A53T mice and WT mice. **F** Quantitative analysis of the level of p-Rab10 in THP-1 cells treated with RBC-EVs from A53T mice and WT mice. **G** Quantitative analysis of *LRRK2* mRNA level in THP-1 cells treated with RBC-EVs from A53T mice and WT mice. **H** Quantitative analysis of *IL1b*, *IL6* and *TNF* mRNA levels using qPCR, in THP-1 cells pretreated with 100 nM Mli2 at resting state (blue) and stimulated with LPS (red). **I** Quantitative analysis of pro-inflammatory cytokines IL-1β, IL-6, TNF-α, IL-8, IFN-γ, IL-2, and IL-12p70, and **J** anti-inflammatory cytokines IL-4, IL-10, and IL-13 using MSD, released by THP-1 cells pretreated with 100 nM Mli2 at resting state (blue) and stimulated with LPS for 24 h (red). *N* = 3 independent experiments in each group. Values are means ± S.E.M, *t* test (**B**), one-way ANOVA test (**D**, **F**, **G**), two-way ANOVA with Bonferroni’s post hoc test (**H**–**J**). *, *P* < 0.05; **, *P* < 0.01; ***, *P* < 0.001; ****, *P* < 0.0001

Next, we investigated whether LRRK2, a kinase enzyme known to be critical to immune regulation of PD patients, and its substrate Rab10, are involved in monocyte sensitization induced by A53T RBC-EVs. As shown in Fig. 6A, B, the levels of LRRK2 and phosphorylated Rab10 (p-Rab10) were significantly higher in A53T mouse monocytes compared to WT monocytes. THP-1 cells cultured with A53T RBC-EVs, but not WT RBC-EVs, also resulted in increased levels of LRRK2 and p-Rab10 (Fig. 6C–G). An inhibitor of LRRK2, Mli2 [48, 49], was used to determine if LRRK2 activity is essential in hyperactivation of monocytes. As observed in Fig. 6H–J, the treatment of Mli2 blocked increased mRNA levels of *IL1b*, *IL6*, and *TNF* and protein levels of pro-inflammatory cytokines (such as IL-1β, IL-6, IL-8 and TNF-α) in THP-1 cells treated with A53T RBC-EVs at both baseline and LPS stimulated state. These results indicate that hyperactivation of monocytes induced by A53T RBC-EVs is mediated at least in part by LRRK2 kinase activity.

### Confirmation of sensitization of monocytes in PD patients

**Fig. 7:**
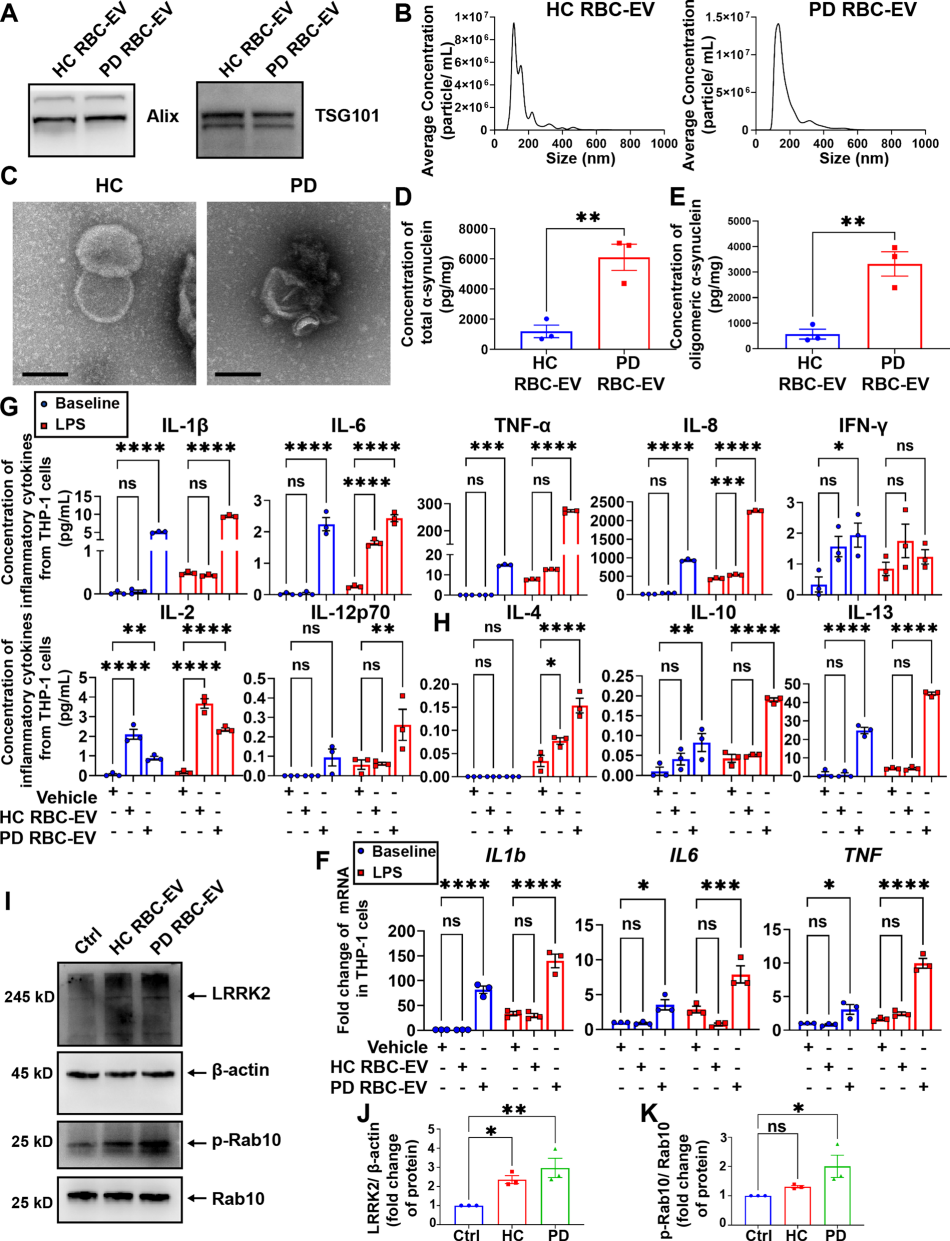
RBC-EVs from PD patients induced immune sensitization of THP-1 cells.** A** Western blot to assess the presence of EV markers Alix and TSG101 in RBC-EV lysates from PD patients and healthy controls. **B** Nanoparticle trafficking analyzed the size distribution and concentration of RBC-EVs from PD patients and healthy controls. **C** Representative electron micrograph images of RBC-EVs from PD patients and healthy controls. Scale bar, 500 nm. **D** Levels of total α-syn in RBC-EVs from PD patients and healthy controls. **E** Levels of oligomeric α-syn in RBC-EVs from PD patients and healthy controls. **F** Quantitative analysis of *IL1b*, *IL6* and *TNF* mRNA levels using qPCR, in THP-1 cells pretreated with RBC-EVs from PD patients and healthy controls at resting state (blue) and stimulated with LPS for 24 h (red). **G** Quantitative analysis of pro-inflammatory cytokines IL-1β, IL-6, TNF-α, IL-8, IFN-γ, IL-2, and IL-12p70, and **H** anti-inflammatory cytokines IL-4, IL-10, and IL-13 using MSD, released by THP-1 cells pretreated with RBC-EVs from PD patients and healthy controls at resting state (blue) and stimulated with LPS for 24 h (red). **I** Western blot to assess the levels of LRRK2 and p-Rab10 in THP-1 cells treated with RBC-EVs from PD patients and healthy controls. **J** Quantitative analysis of the levels of LRRK2 in THP-1 cells treated with RBC-EVs from PD patients and healthy controls. **K** Quantitative analysis of the levels of p-Rab10 in THP-1 cells treated with RBC-EVs from PD patients and healthy controls. *N* = 3 independent experiments in each group. Values are means ± S.E.M, *t* test (**D**, **E**), one-way ANOVA test (**J**, **K**), two-way ANOVA with Bonferroni’s post hoc test (**F**–**H**). *, *P* < 0.05; **, *P* < 0.01; ***, *P* < 0.001; ****, *P* < 0.0001

To confirm the significance of the above results in human disease, RBC-EVs were isolated from cultured RBCs from 5 PD patients and 3 neurologically normal control subjects (Table 1 and Fig. 7A–C). Consistent with earlier observations [30, 31, 34, 35], total and oligomeric α-syn contained in RBC-EVs were significantly higher in PD patients compared to healthy controls (HCs) (Fig. 7D, E). In addition, consistent with A53T mouse experiments, THP-1 cells treated with the RBC-EVs derived from PD patients demonstrated significantly elevated mRNA levels of *IL1b, IL6 and  TNF* (Fig. 7F), and concentration of inflammatory cytokines (Fig. 7G, H) in THP-1 cells at resting state. Following 24 h LPS stimulation, the mRNA and protein levels of inflammatory cytokines further significantly increased in THP-1 cells pre-treated with PD RBC-EVs (Fig. 7F–H). In addition, LRRK2 and p-Rab10 expression were significantly increased in THP-1 cells cultured with PD RBC-EVs, while LRRK2 and p-Rab10 were only slightly increased in THP-1 cells treated with HC RBC-EVs (Fig. 7I–K). These results indicate that RBC-EVs derived from PD patients are able to induce inflammatory sensitization of monocytes, with a process likely involving LRRK2 activation.

**Fig. 8:**
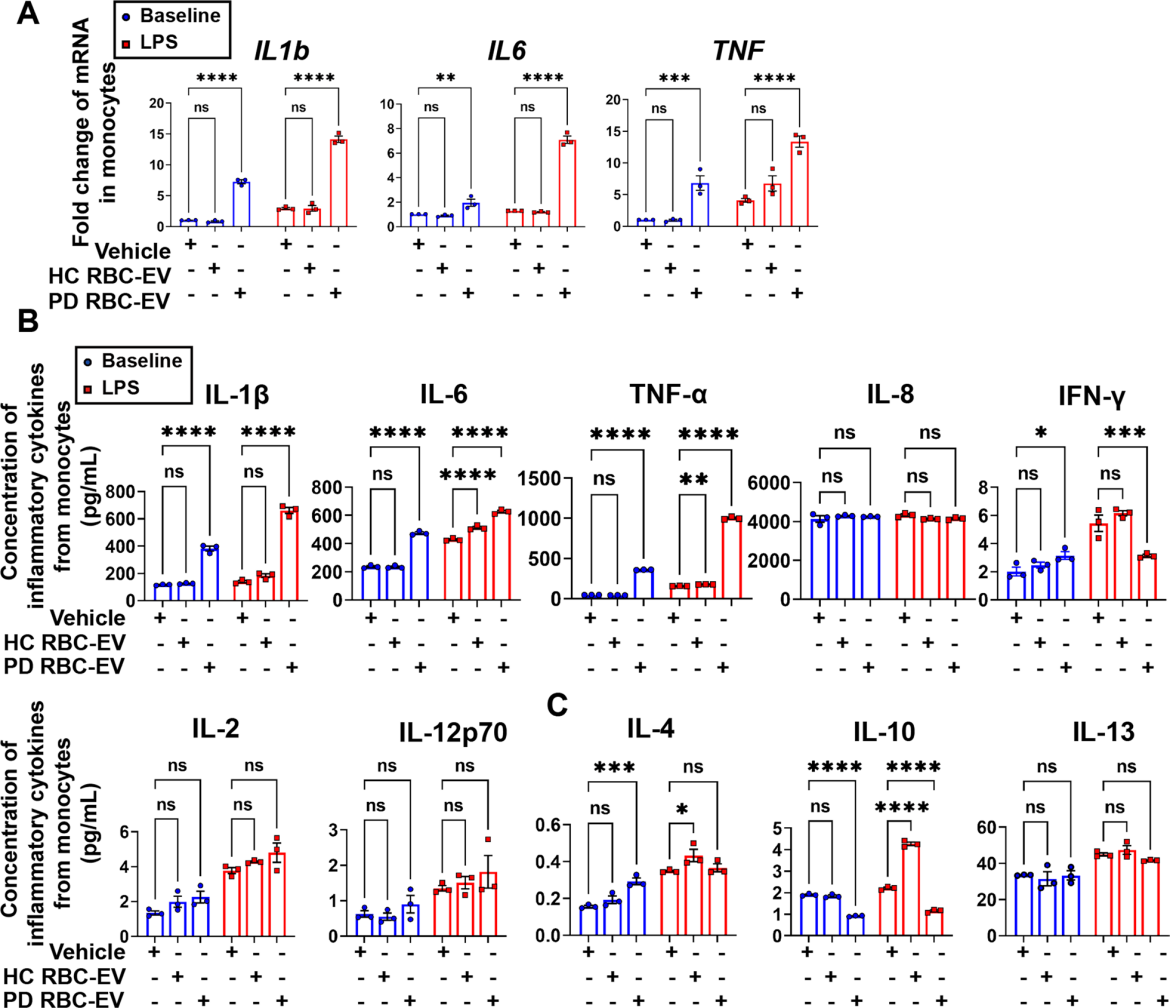
Inflammatory sensitization of monocytes induced by RBC-EVs from PD patients.** A** Quantitative analysis of *IL1b*, *IL6* and *TNF* mRNA levels using qPCR, in monocytes pretreated with RBC-EVs from PD patients and healthy controls at resting state (blue) and stimulated with LPS for 24 h (red). **B** Quantitative analysis of pro-inflammatory cytokines IL-1β, IL-6, TNF-α, IL-8, IFN-γ, IL-2, and IL-12p70, and **C** anti-inflammatory cytokines IL-4, IL-10, and IL-13 using MSD, released by monocytes pretreated with RBC-EVs from PD patients and healthy controls at resting state (blue) and stimulated with LPS for 24 h (red). *N* = 3 independent experiments in each group. Values are means ± S.E.M, two-way ANOVA with Bonferroni’s post hoc test. *, *P* < 0.05; **, *P* < 0.01; ***, *P* < 0.001; ****, *P* < 0.0001

To further confirm that PD RBC-EVs can induce the hyperactivation of monocytes, monocytes from healthy controls were isolated and treated with HC RBC-EVs or PD RBC-EVs. As shown in Fig. 8, PD RBC-EVs can significantly increase the mRNA levels of *IL1b*, *IL6* and *TNF*, and the protein levels of pro-inflammatory cytokines, such as IL-1β, IL-6, TNF-α, and IFN-γ in monocytes. Following 24 h LPS stimulation, the mRNA levels of *IL1b*, *IL6* and *TNF* and the protein level of pro-inflammatory cytokines, especially IL-1β, IL-6 and TNF-α, became significantly higher in monocytes pre-treated with PD RBC-EVs than HC RBC-EVs.

## Discussion

The major observations of the current study centered on hyperactivation of monocytes of PD patients and related mechanisms. In our previous study, we showed that oligomeric α-syn containing RBC-EVs can traffic across the BBB via an adsorptive-mediated transcytosis process and activate microglia in the CNS [8]. Considering the direct exposure of RBC-EVs to circulating monocytes, we hypothesized that RBC-EVs may be involved in the hyperactivation of monocytes in PD patients whose inflammatory markers are commonly elevated in blood [21]. We demonstrated that monocytes obtained from PD patients and A53T mice both exhibited higher production of mRNA of inflammatory cytokine genes and higher levels of secreted cytokines at baseline state compared to controls, and produced significantly more inflammatory cytokines after LPS stimulation, which was consistent with several previous reports [24, 25]. More importantly, RBC-EVs derived from both PD patients and A53T mice, but not healthy controls and SNCA KO mice, were able to induce hyperactivation of THP-1 cells and monocytes in vitro. Moreover, at resting or stimulated state, the alterations of inflammatory cytokines in monocytes treated with PD RBC-EVs were quite consistent with the results in THP-1 cells, indicating that α-syn containing RBC-EVs likely participate in the hyperactivation of monocytes in PD patients.

In whole blood, α-syn species exist either in free forms or in association with EVs. In particular, RBCs can be viewed as a reservoir of α-syn and are able to secrete EVs harboring different structural forms of α-syn [6, 7, 35, 50]. Previously, we have demonstrated that RBC-EVs derived from PD patients and PD mice contain more pathological α-syn and can cross the BBB and deposit α-syn in astrocytes and microglia, resulting in astrocytic dysfunction and microglial activation [7, 8]. To characterize the pathophysiological relevance of α-syn in RBC-EVs-driven hyperactivation of monocytes, the immune regulation of oligomeric α-syn containing RBC-EVs on monocytes was investigated at resting baseline and LPS stimulated states. In the absence of LPS stimulation, A53T RBC-EVs could hyperactivate the resting THP-1 cells, resulting in increased cytokine expression and release. Following LPS stimulation, the production of inflammatory cytokines in THP-1 cells further increased after pre-treatment with α-syn containing A53T RBC-EVs or PD RBC-EVs. These results demonstrate that the oligomeric α-syn containing RBC-EVs are involved in hyperactivation of monocytes in PD. We further directly examined the effects of both monomeric and oligomeric α-syn on monocytes and found that oligomeric α-syn can activate THP-1 cells much more potently than monomeric α-syn. More importantly, A53T RBC-EVs elicited a more potent immune response in monocytes compared to the free form of oligomeric α-syn. By normalizing LPS stimulated to the baseline state levels, the fold change of pro-inflammatory cytokines (e.g., IL-1β, IL-8, and TNF-α) in THP-1 cells pre-treated with A53T RBC-EVs was significantly higher than THP-1 cells pre-treated with free oligomeric α-syn (Additional file 1: Fig. S5). Previous studies reported that free oligomeric α-syn can bind toll-like receptor 2 or 4 (TLR2 or TLR4) on microglia to induce the release of pro-inflammatory cytokines in the CNS [51–53], suggesting that a similar mechanism may be potentially involved in the interaction between oligomeric α-syn containing RBC-EVs with monocytes. However, the current study suggests that it is the α-syn containing RBC-EVs, not the free forms of α-syn, that most effectively sensitize monocytes. Thus, α-syn containing RBC-EVs may be an essential factor to the hyperactivation of monocytes in PD.

We tested the hypothesis that RBC-EV-driven immune sensitization involves endocytosis using three different endocytosis inhibitors (Me-β-CD, Nocodazole and Amiloride) and found that Me-β-CD and Nocodazole had potent inhibitory effects. Me-β-CD is a cholesterol depleting agent that can inhibit both clathrin- and caveolae-mediated endocytosis [33, 54, 55], while Nocodazole is a microtubule polymerization blocker that prevents the trafficking of early endosomes to lysosomes [44, 56]. On the other hand, Amiloride, which inhibits macropinocytosis by lowering submembranous pH by interfering Na^+^/H^+^ exchange [45], had a weaker inhibitory effect on immune sensitization. Taken together, these data suggest that receptor-mediated endocytosis plays a key role in modulating oligomeric α-syn-containing RBC-EVs sensitization of monocytes and it is likely to involve interaction with receptors that are enriched in clathrin- and/or caveolae coated pits. Of note, our study does not exclude the possibility that α-syn-containing RBC-EVs also interact with a surface receptor, e.g., TLR, to mediate the sensitization of monocytes. This is particularly important to the engagement of surface receptors that are critical to the downstream activation of LRRK2.

Mutations in *LRRK2* gene are the most common cause of familial types of PD and polymorphisms in *LRRK2* have been shown to modulate risk for sporadic PD [3, 4, 36, 57]. In the periphery, LRRK2 is expressed in myeloid cells, including monocytes and macrophages [58, 59]. Recent works have revealed that PD patients exhibited increased LRRK2 protein expression and kinase activity in hyperactivated immune cells [37–40, 60, 61], which is consistent with our observations in both PD patients and A53T mice monocytes. We showed that treatment of THP-1 monocytes with PD RBC-EVs also resulted in increased expression and kinase activity of LRRK2. Furthermore, by inhibiting the kinase activity of LRRK2 with Mli-2 [48, 49], the hyperactivation of monocytes was significantly relieved, indicating the crucial role of LRRK2 in the PD RBC-EVs-driven immune dysregulation process. This data set is consistent with reports, where knocking out LRRK2 or inhibiting the kinase activity of LRRK2 can attenuate the neuroinflammation induced by α-syn [58, 62]. Although the endocytosis of α-syn containing RBC-EVs and increased kinase activity of LRRK2 have been proved to be highly associated with the hyperactivation of PD monocytes, the precise mechanisms involved in the hyperactivation of PD monocytes, including those beyond endocytosis of RBC-EVs and kinase of LRRK2, need to be investigated further.

In the context of neurological disorders, such as PD, it is thought that the initial immune response may be protective, but chronic inflammation can contribute to the onset and progression of disease [63]. In our study, anti-inflammatory cytokines (such as IL-10), were increased along with pro-inflammatory cytokines (such as IL-1β, IL-6, and TNF-α) in PD patients, a phenomenon also reported in other investigations [21, 26, 64]. The underlying mechanism is yet to be defined further, but one potential reason could be a compensatory response of monocytes while stressed. Indeed, this protective mechanism to counter increased peripheral inflammation and promote immune responses to cope with exposure to pathological α-syn (e.g., activation of B-cells, production of IFN-γ and phagocytotic ability of monocytes), has been proposed previously [65, 66]. In addition, of note, in our study, the extent of increase in pro-inflammatory cytokines was much higher than anti-inflammatory cytokines (Figs. 1, 3, 7 and 8).

## Conclusions

Our findings demonstrated that RBC-EVs containing pathological oligomeric α-syn causes hyperactivation of circulating monocytes, in a process that requires receptor mediated endocytosis and LRRK2 activation. Our data provides a novel perspective for immune dysregulation in sporadic PD and highlights LRRK2 inhibition in peripheral monocytes as a potential therapeutic target for ameliorating PD pathogenesis.

## Supplementary Information

**Additional file 1.** Additional figures.

## Data Availability

All data generated or analyzed during this study are included in this published article and its additional information files.

## Abbreviations

PD: Parkinson’s disease
α-syn: α-Synuclein
CNS: Central nervous system
EV: Extracellular vesicle
RBC-EVs: RBC-derived extracellular vesicles
BBB: Blood–brain barrier
LRRK2: Leucine-rich repeat kinase 2
