# Supplementary material for: α-Synuclein-containing erythrocytic extracellular vesicles: essential contributors to hyperactivation of monocytes in Parkinson’s disease
Source: J Neuroinflammation. 2022 Feb 22;19:53. doi: 10.1186/s12974-022-02413-1 (PMC8862590; doi:10.1186/s12974-022-02413-1)
Supplement: Supplementary file 1 — Additional file 1. Additional figures. [file 12974_2022_2413_MOESM1_ESM.docx]

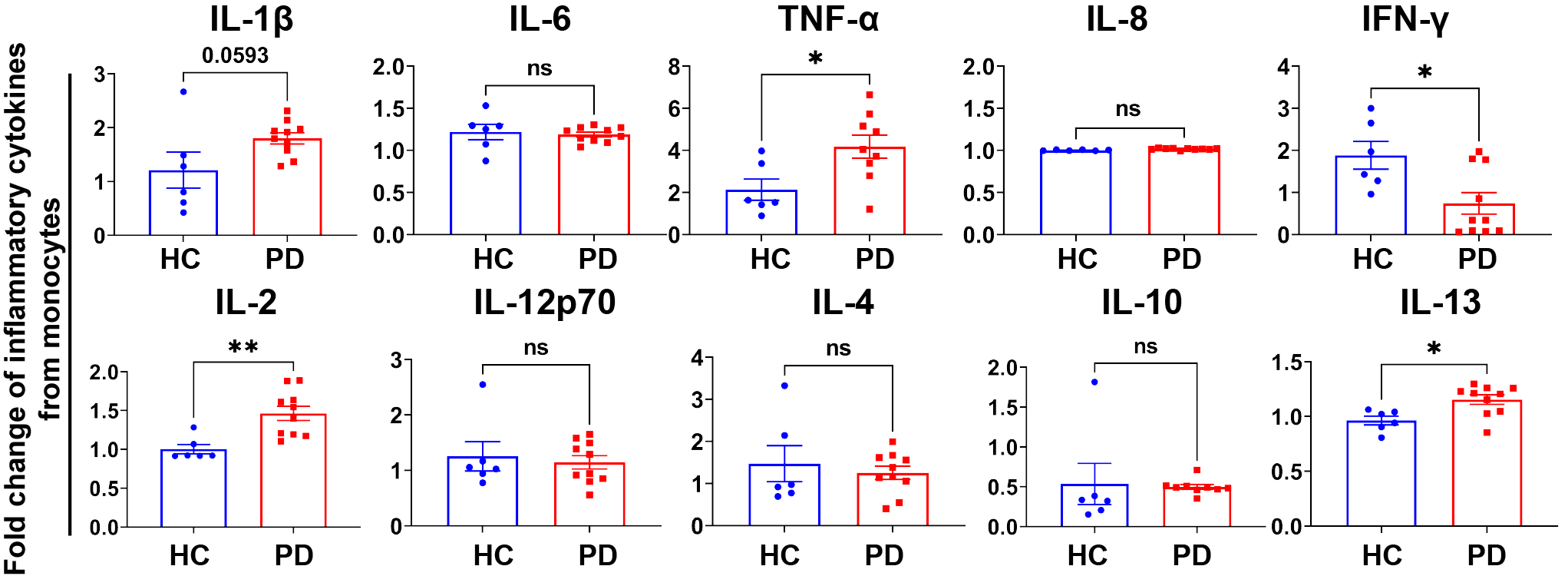


**Figure S1 Fold change of inflammatory cytokines after LPS stimulation in monocytes.** Comparison of pro-inflammatory cytokines IL-1β, IL-6, TNF-α, IL-8, IFN-γ, IL-2, IL-12p70, and anti-inflammatory cytokines IL-4, IL-10, IL-13 released by monocytes of PD patients and healthy controls. The fold change was calculated by normalizing LPS stimulated cytokine levels to resting state cytokine levels. N=6 independent human samples in health control group and N=10 independent human samples in PD group. Values are means ± S.E.M, t-test. *, *P* <0.05; **, *P*< 0.01.


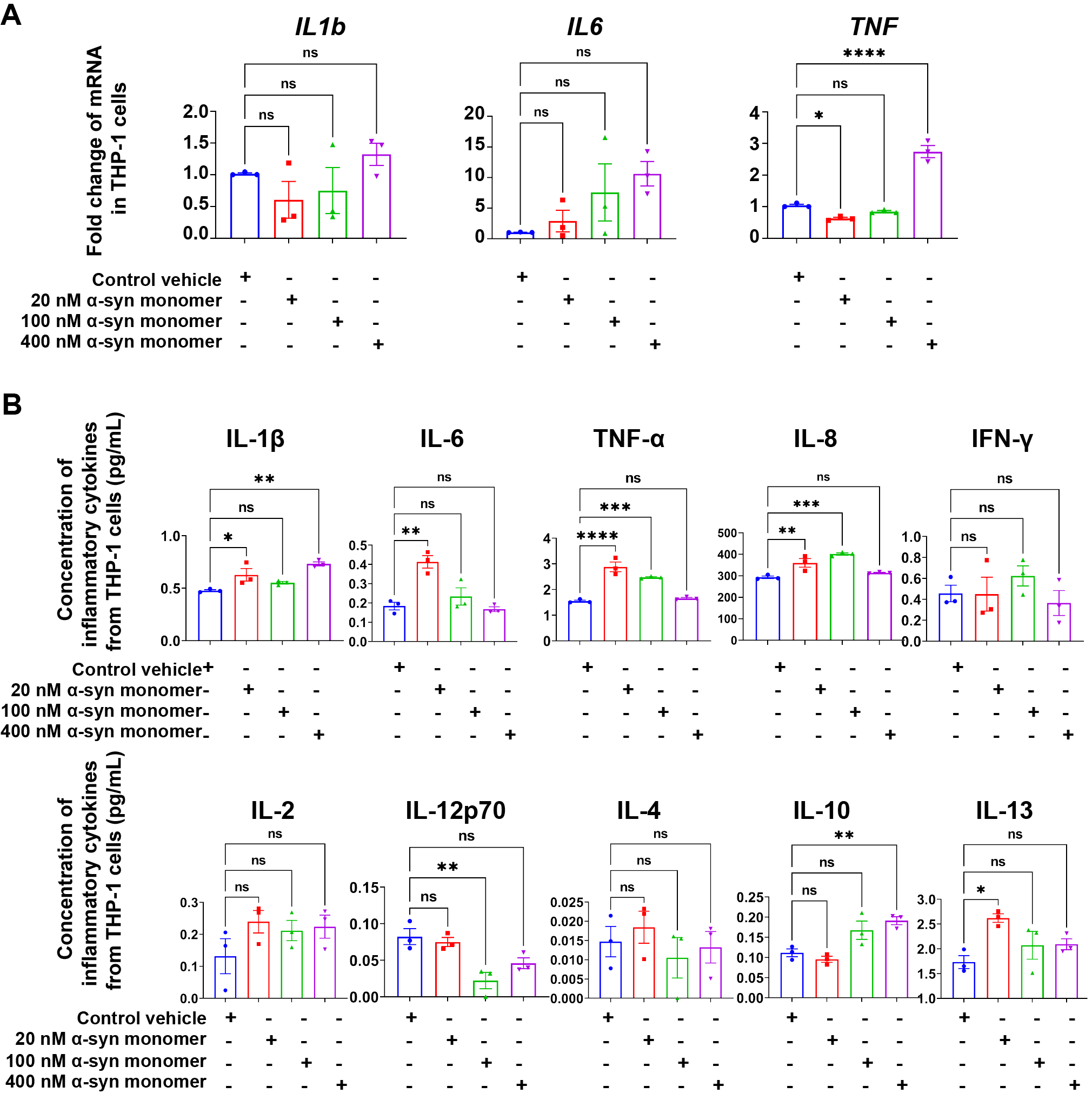


**Figure S2** **Monomeric α-syn weakly induced immune sensitization of THP-1 cells. (A)** Quantitative analysis of *IL1b*, *IL6* and *TNF* mRNA level using qPCR, in THP-1 cells pretreated with monomeric α-syn after LPS stimulation. **(B)** Quantitative analysis of pro-inflammatory cytokines IL-1β, IL-6, TNF-α, IL-8, IFN-γ, IL-2, IL-12p70, and anti-inflammatory cytokines IL-4, IL-10, IL-13 using MSD, released by THP-1 cells pretreated with monomeric α-syn after LPS stimulation. N=3 independent experiments in each group. Values are means ± S.E.M, one-way ANOVA test. *, *P* <0.05; **, *P*< 0.01; ***, *P*< 0.001; ****, *P*< 0.0001.


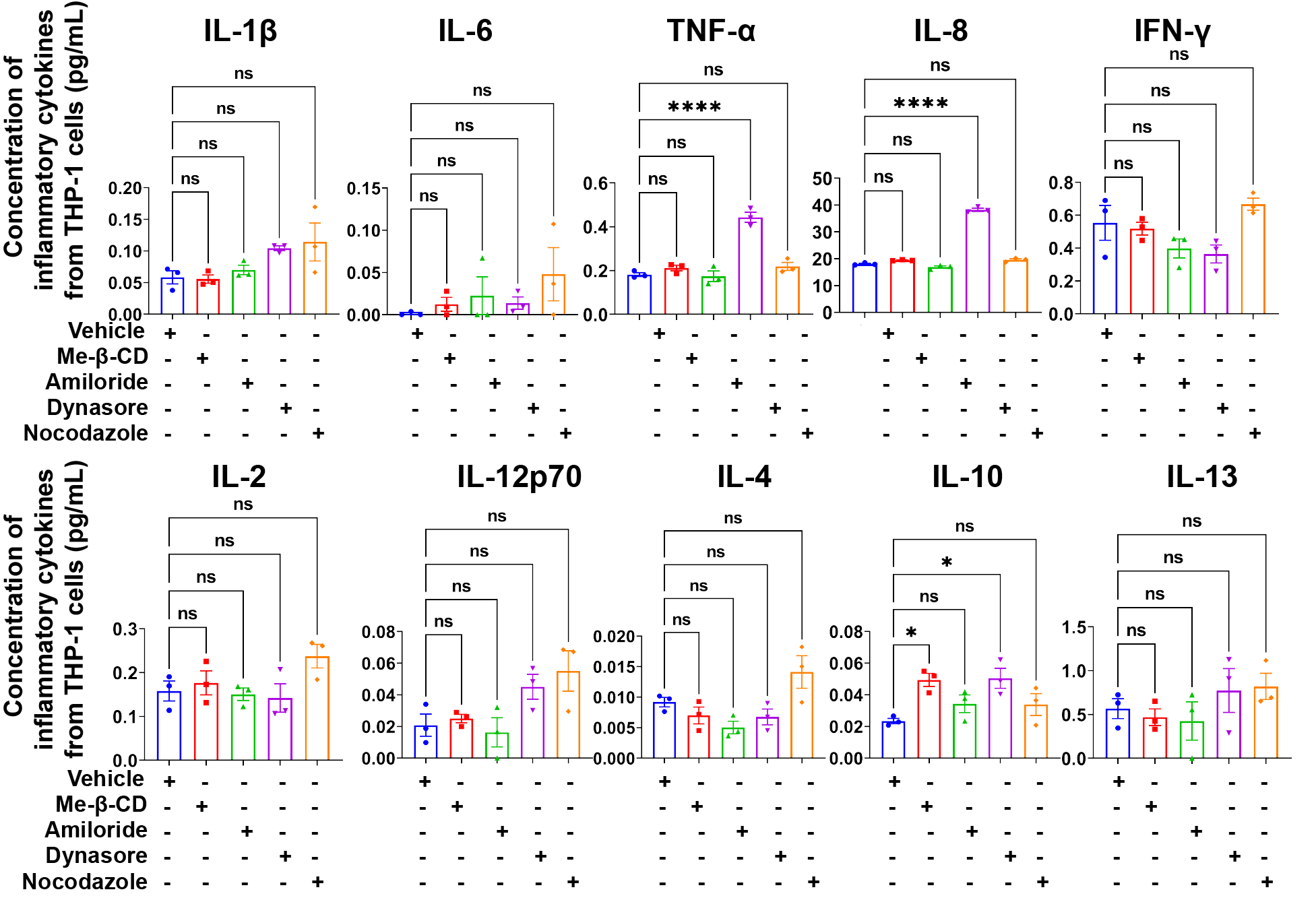
**Figure S3** **The effect of four endocytosis inhibitors on monocyte hyperactivation.** Quantitative analysis of pro-inflammatory cytokines IL-1β, IL-6, TNF-α, IL-8, IFN-γ, IL-2, IL-12p70, and anti-inflammatory cytokines IL-4, IL-10, IL-13 using MSD, released by THP-1 cells pretreated with Dynasore, Nocodazole, Me-β-CD and Amiloride after LPS stimulation. N=3 independent experiments in each group. Values are means ± S.E.M, one-way ANOVA test. *, *P* <0.05; **, *P*< 0.01; ***, *P*< 0.001; ****, *P*< 0.0001.


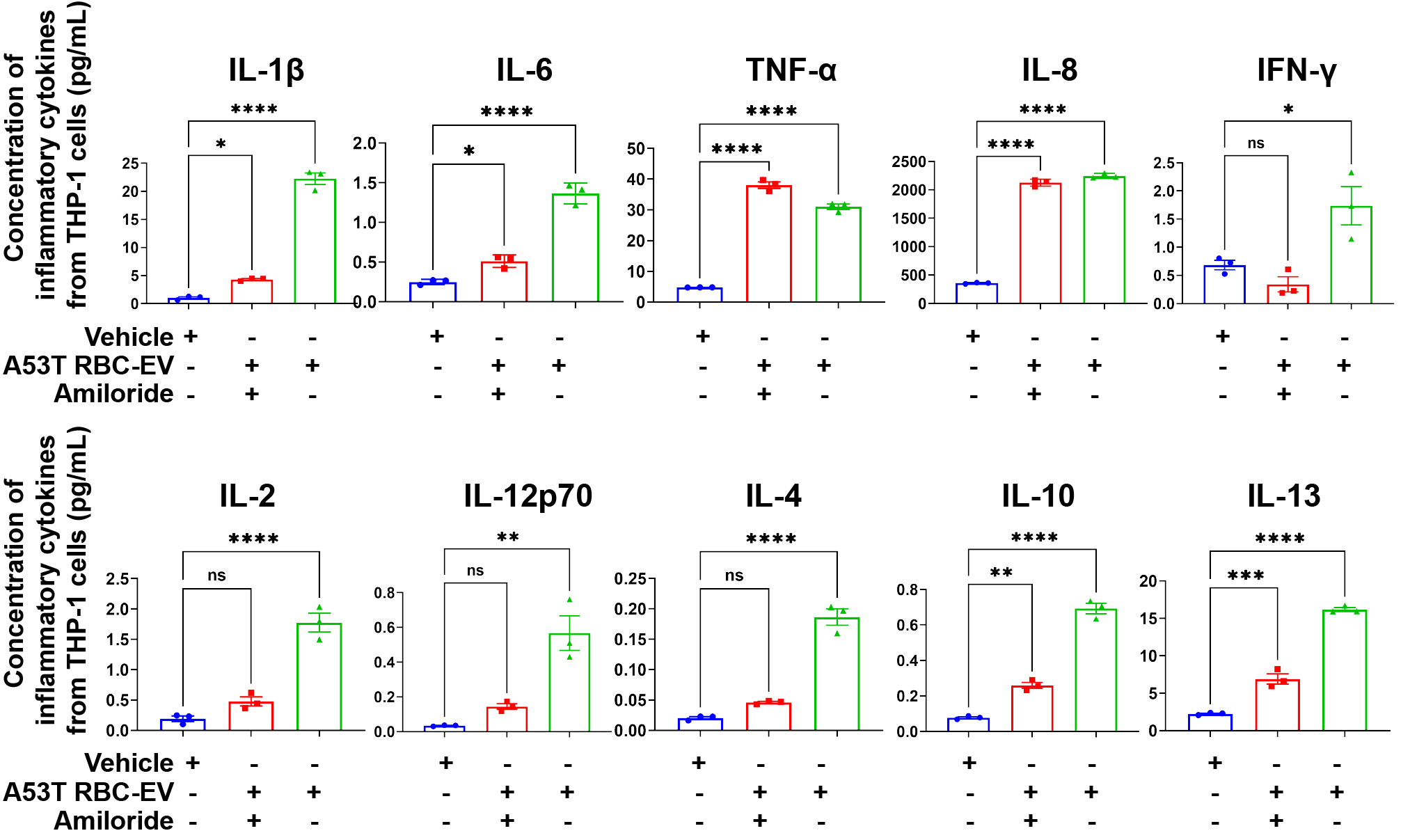


**Figure S4. Amiloride reduced the immune sensitization of monocytes induced by A53T RBC-EVs.** Quantitative analysis of pro-inflammatory cytokines IL-1β, IL-6, TNF-α, IL-8, IFN-γ, IL-2, IL-12p70, and anti-inflammatory cytokines IL-4, IL-10, IL-13 using MSD, released by THP-1 cells pretreated with PD RBC-EVs, along with Amiloride, after LPS stimulation. N=3 independent experiments in each group. Values are means ± S.E.M, one-way ANOVA test. *, *P* <0.05; **, *P*< 0.01; ***, *P*< 0.001; ****, *P*< 0.0001.


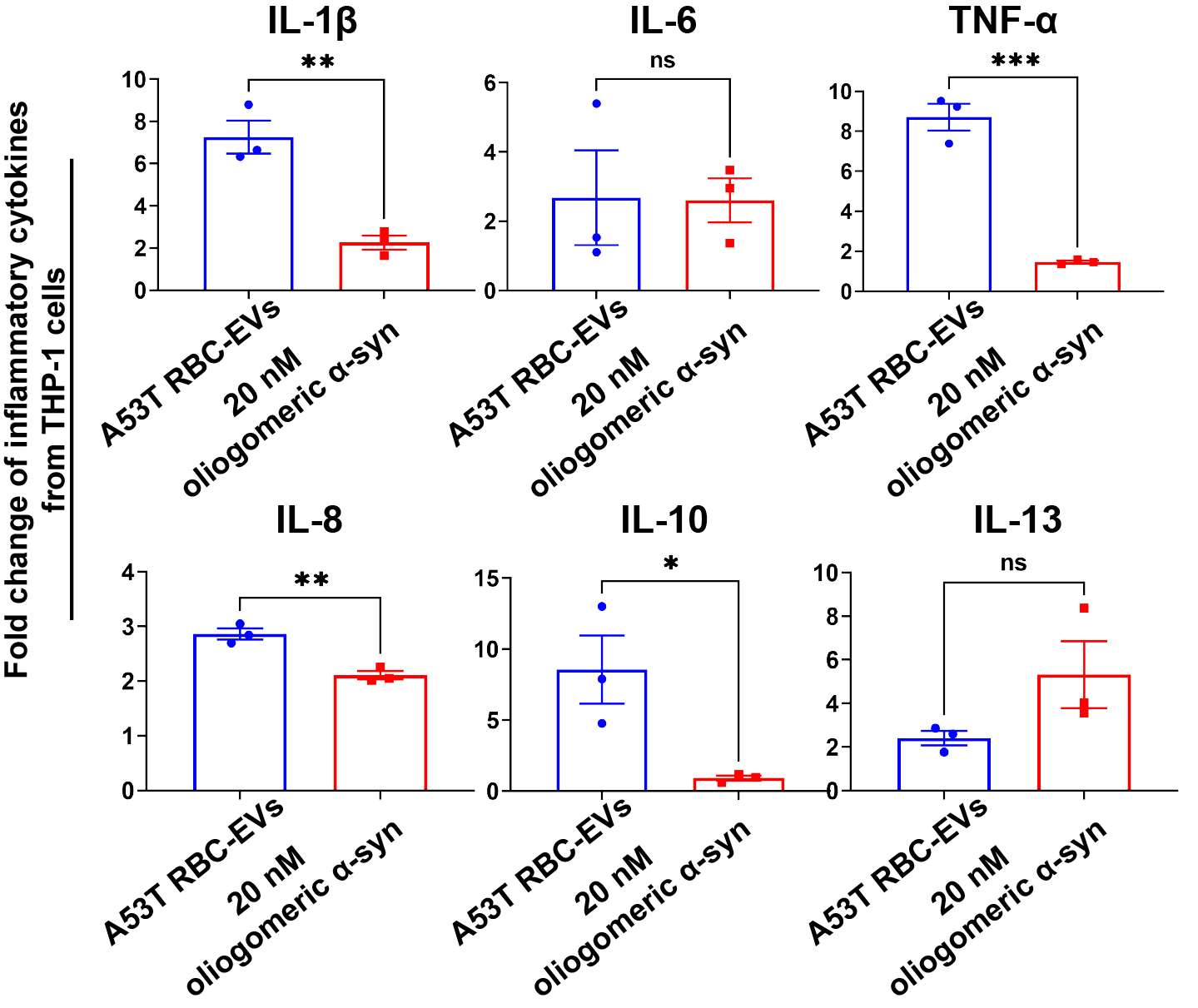


**Figure S5 More potent immune response in THP-1 monocytes elicited by A53T RBC-EVs.** Comparison of pro-inflammatory cytokines IL-1β, IL-6, IL-8, TNF-α, and anti-inflammatory cytokines IL-10 and IL-13, released by THP-1 cells pre-treated with A53T RBC-EVs (blue) or 20 nM oligomeric α-syn (red). The fold change was calculated by normalizing LPS stimulated cytokine levels to resting state cytokine levels in **Figure 3** and **Figure 4**. N=3 independent experiments in each group. Values are means ± S.E.M, t test. *, *P* <0.05; **, *P*< 0.01, ***, *P*< 0.001.

**Full-length Western blots：**


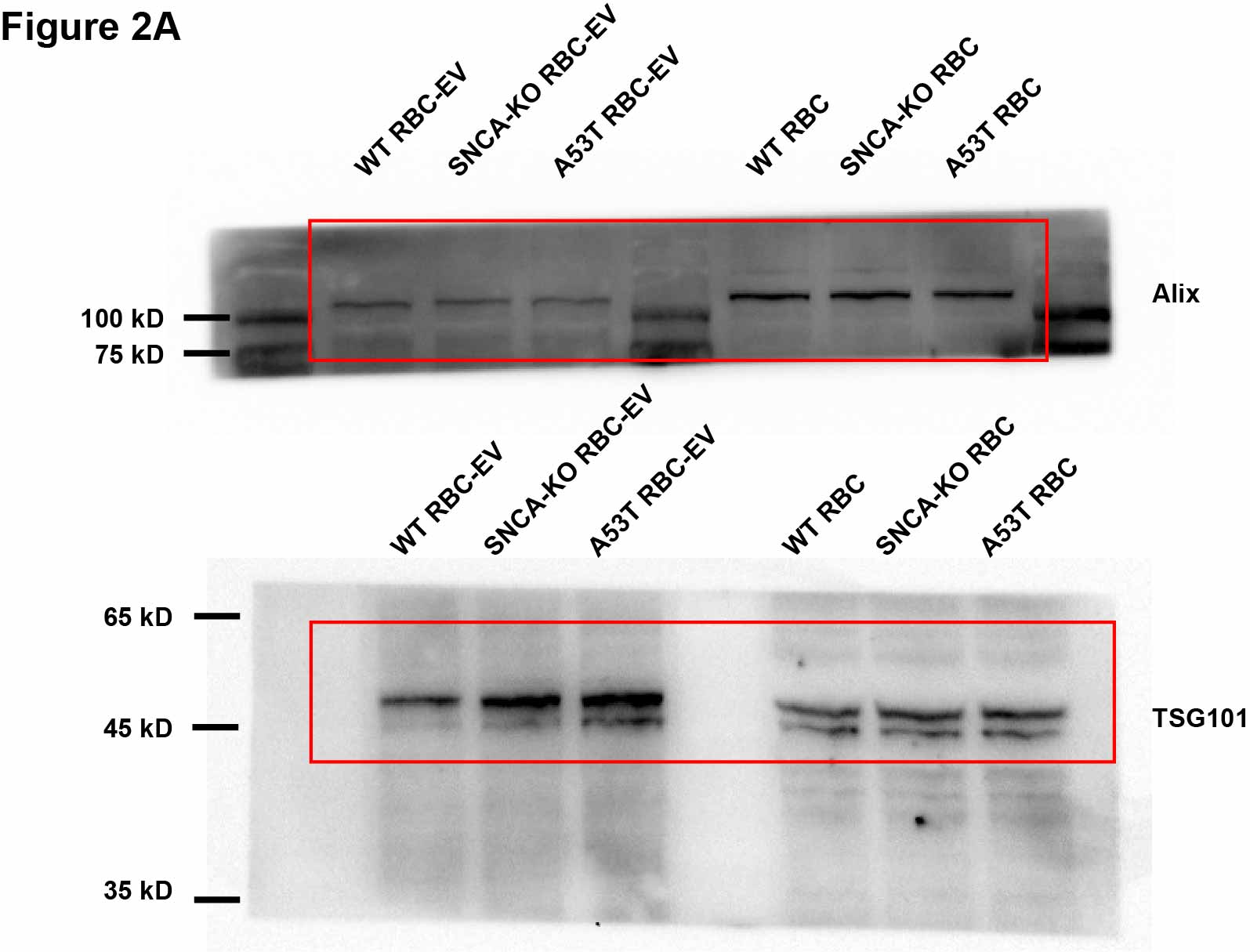


PVDF membranes were separated into two strips for blotting with anti-Alix, anti-TSG101, respectively. The strips from the same gel/membrane were exposed and imaged at the same time. *Red boxes* indicate the cropped images shown in **Fig. 2A**.


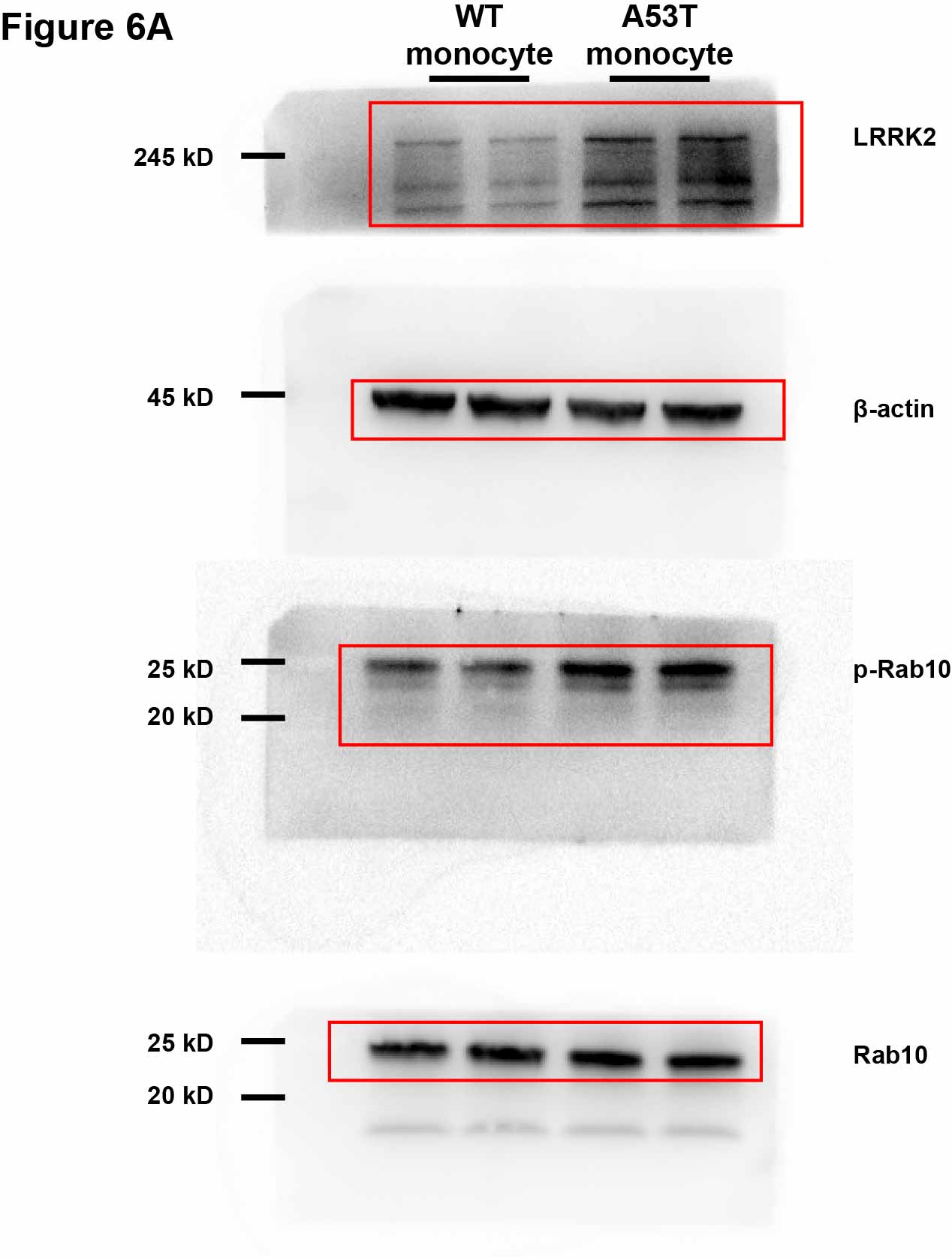


PVDF membranes were separated into three strips for blotting with anti-LRRK2, anti-β-actin, anti-p-Rab10 respectively. The strip of p-Rab10 was stripped by stripping buffer and blotted with anti-Rab10. The strips from the same gel/membrane were exposed and imaged at the same time. *Red boxes* indicate the cropped images shown in **Fig. 6A**.


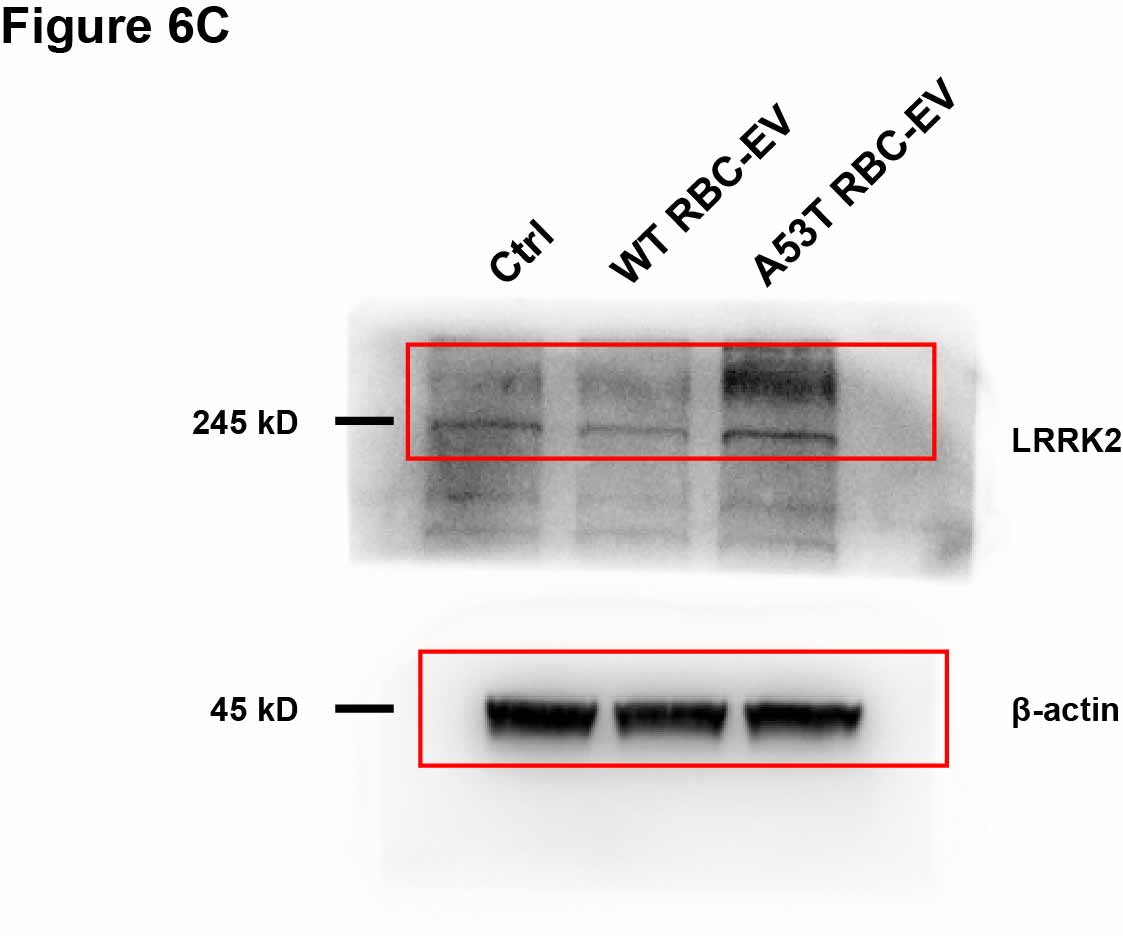


PVDF membranes were separated into two strips for blotting with anti-LRRK2, anti-β-actin, respectively. The strips from the same gel/membrane were exposed and imaged at the same time. *Red boxes* indicate the cropped images shown in **Fig. 6C**.


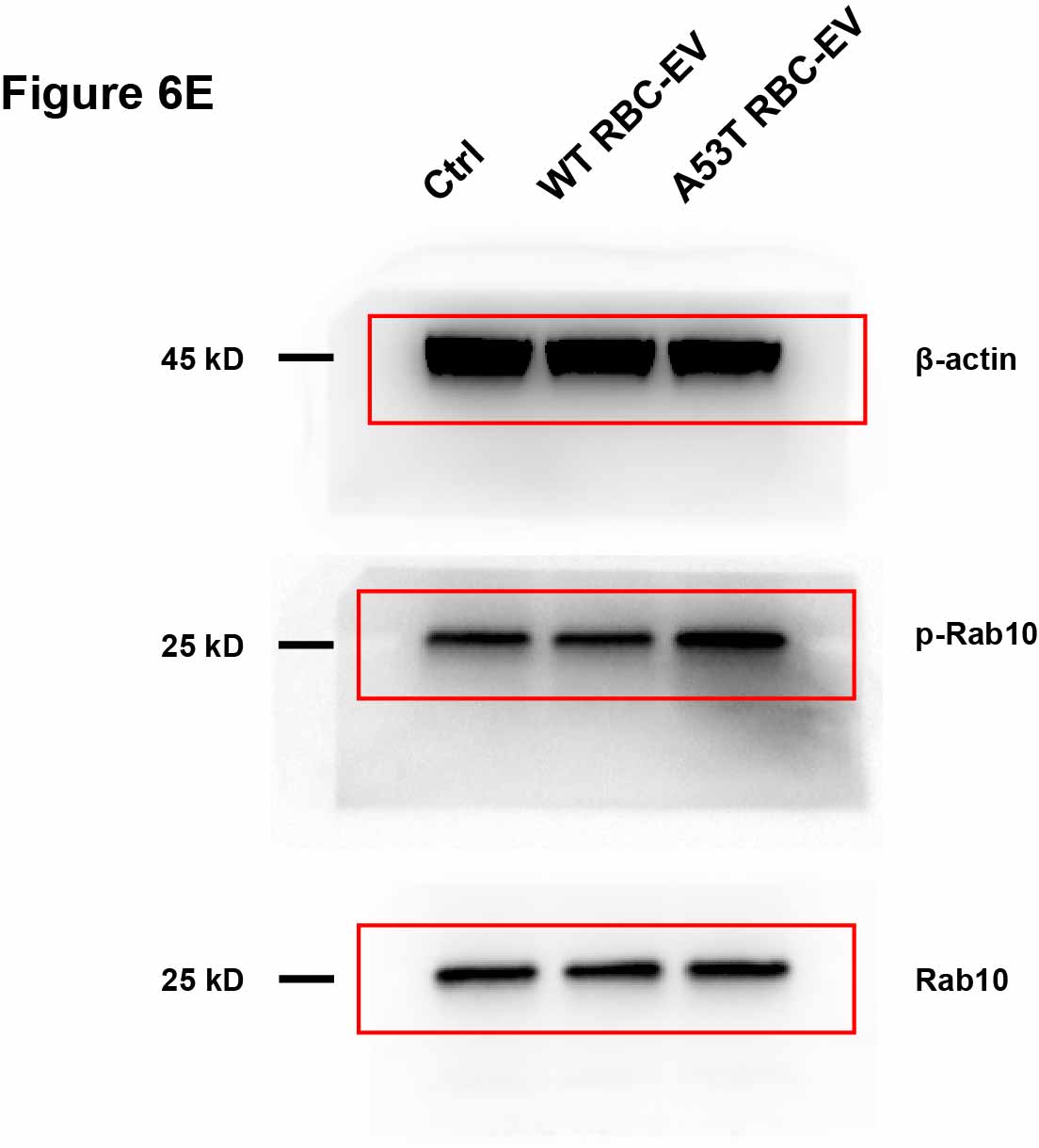


PVDF membranes were separated into two strips for blotting with anti-β-actin and anti-p-Rab10 respectively. The strip of p-Rab10 was stripped by stripping buffer and blotted with anti-Rab10. The strips from the same gel/membrane were exposed and imaged at the same time. *Red boxes* indicate the cropped images shown in **Fig. 6E**.


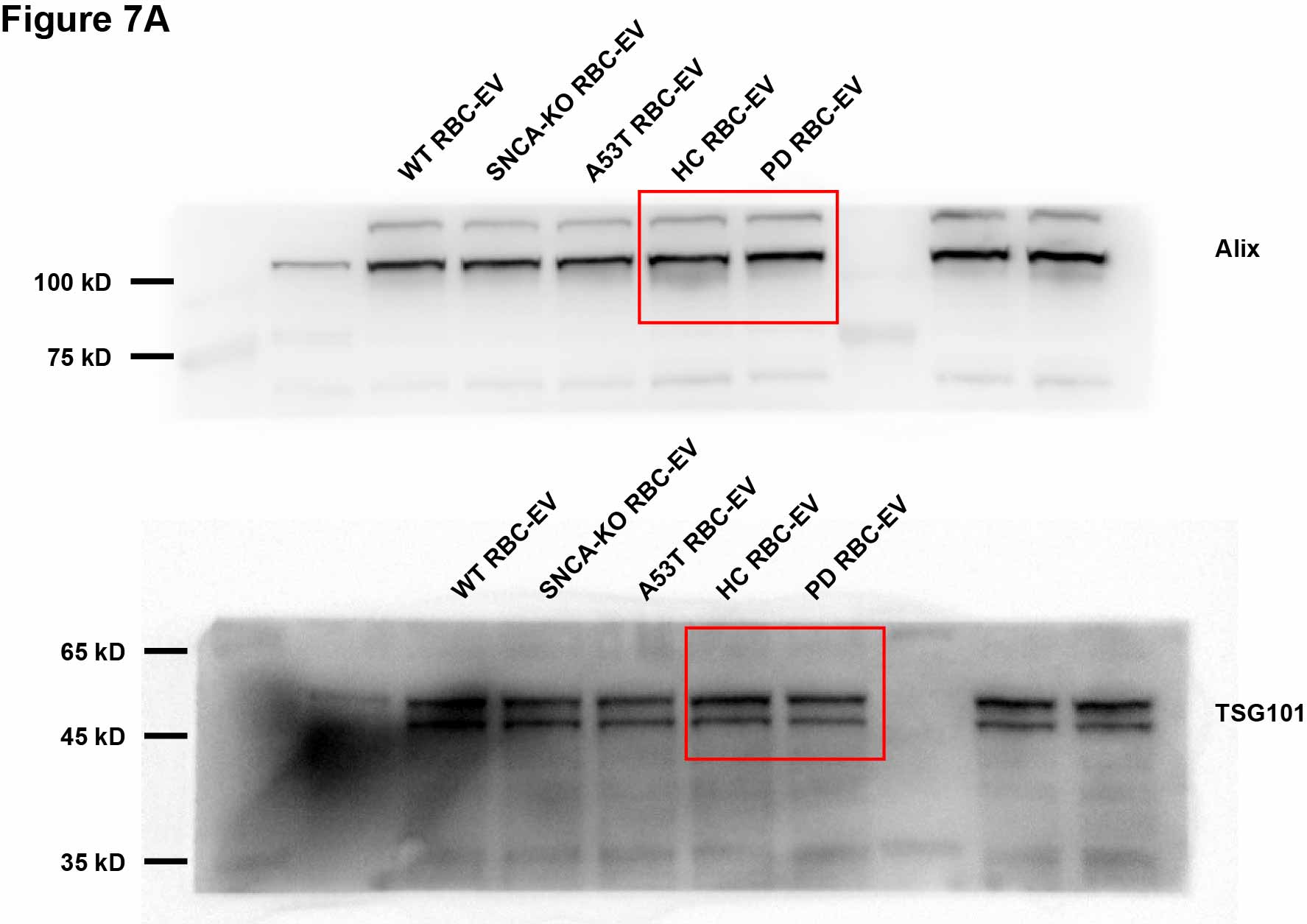


PVDF membranes were separated into two strips for blotting with anti-Alix, anti-TSG101, respectively. The strips from the same gel/membrane were exposed and imaged at the same time. *Red boxes* indicate the cropped images shown in **Fig. 7A**.


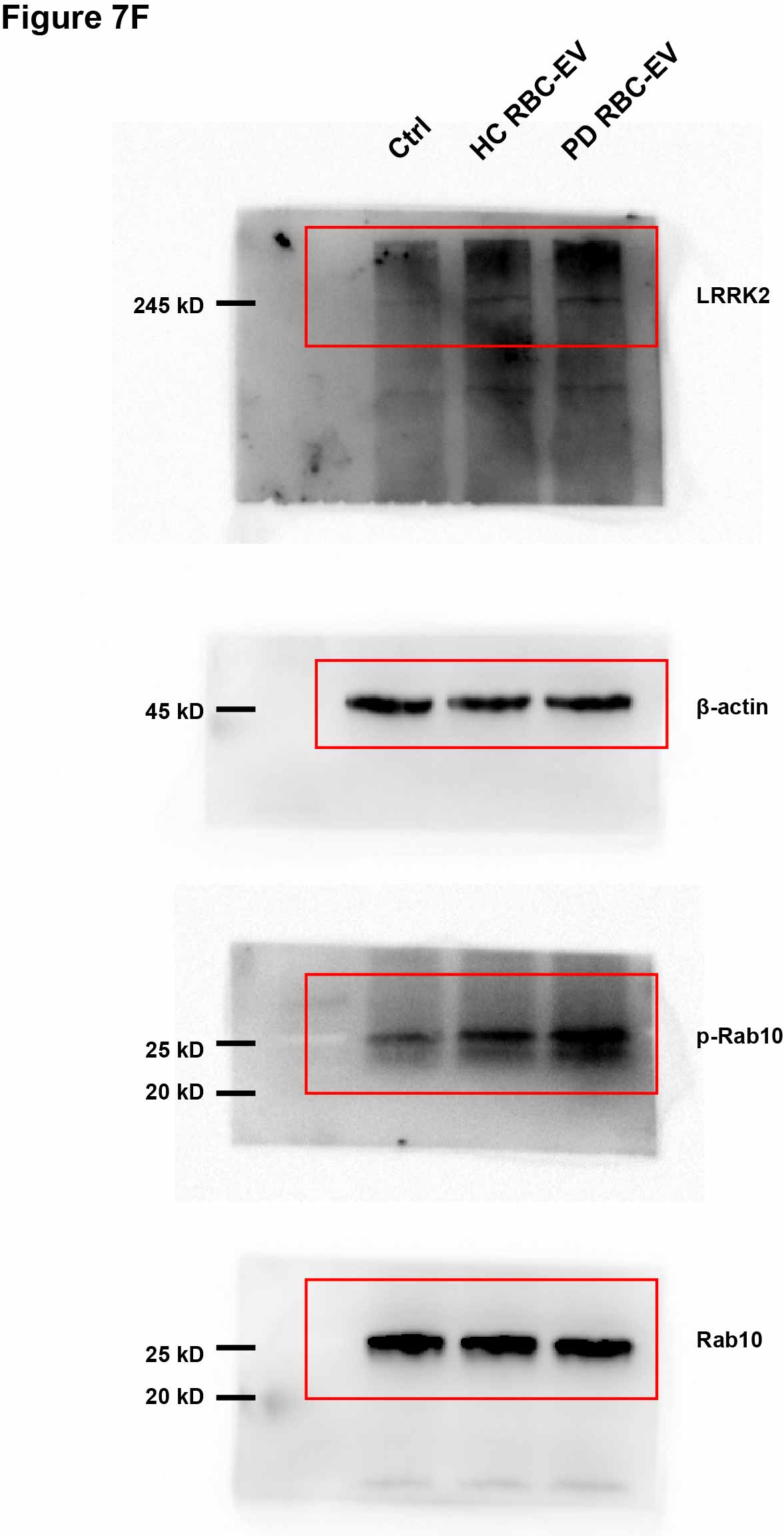


PVDF membranes were separated into three strips for blotting with anti-LRRK2, anti-β-actin, anti-p-Rab10 respectively. The strip of p-Rab10 was stripped by stripping buffer and blotted with anti-Rab10. The strips from the same gel/membrane were exposed and imaged at the same time. *Red boxes* indicate the cropped images shown in **Fig. 7F**.
